# Continuous Subcutaneous Insulin Infusion Versus Multiple Daily Injections for Glycemic Management in Pregnant Women With Type 1 Diabetes: A Systematic Review and Meta-Analysis

**DOI:** 10.1016/j.aed.2025.08.005

**Published:** 2025-08-21

**Authors:** Angie Carolina Alonso Ramírez, Jonathan Víctor Salazar Ore, Olivia Murga, Stephani Carolina Salvatierra Moreno, Hugo Lana Devita, Maigualida Nieto, Jobby John, Loura Aly, Gabriela D. Briceno Silva, Stephin Zachariah Saji, Victor Sebastian Arruarana, Ricardo Correa Marquez, Ernesto Calderón Martinez

**Affiliations:** 1 Pontificia Universidad Javeriana, Facultad de Medicina, Bogotá, Colombia; 2 Universidad de Buenos Aires, Facultad de Ciencias Médicas, Buenos Aires, Argentina; 3 Faculty of Medicine, Universidad de Los Andes, Mérida, Venezuela; 4 Faculty of Medicine, Universidad Central de Venezuela, Caracas, Venezuela; 5 Faculty of Medicine, Dr. Somervell Memorial CSI Medical College & Hospital, Thiruvananthapuram, India; 6 Faculty of Medicine, Ilia State University, Tbilisi, Georgia; 7 Faculty of Medicine, Universidad de Oriente, Barcelona, Venezuela; 8 Faculty of Medicine, Our Lady of Fatima University College of Medicine, Valenzuela, Philippines; 9 Department of Internal Medicine, Brookdale University Hospital and Medical Center, New York, New York; 10 Department of Endocrinology, Diabetes and Metabolism, Cleveland Clinic, Cleveland, Ohio; 11 Department of Internal Medicine, The University of Texas Health Science Center at Houston, Texas

**Keywords:** pregnant people, insulin, insulin infusion systems, diabetes mellitus, type 1

## Abstract

**Background/Objective:**

To compare glycemic management outcomes between continuous subcutaneous insulin infusion (CSII) and multiple daily injections in pregnancy and assess maternal and neonatal outcomes. There is an ongoing increase in diabetes rates worldwide. The International Diabetes Federation estimated that there were 23.0 million cases of hyperglycemia (19.7%) before and during pregnancy in 2024 worldwide, causing 1.5 million deaths annually. Pregnant women with pregestational type 1 diabetes mellitus (T1DM) struggle with 2-4 times more maternal and fetal complications than pregnant women without diabetes.

**Methods:**

Following the Preferred Reporting Items for Systematic Reviews and Meta-Analyses guidelines, we searched PubMed MEDLINE, Cochrane, Scopus, Web of Science, Embase, Cumulative Index to Nursing and Allied Health Literature, and China National Knowledge Infrastructure through January 2025. Eligible studies included randomized controlled trials, cohort studies, and case-control studies comparing CSII and multiple daily injection in pregnant women with T1DM. Data were pooled using random-effects models, with effect sizes expressed as mean differences (MDs), standardized mean differences, or risk ratios (RRs) with 95% CIs.

**Results:**

Thirty-one studies encompassing 6532 pregnant women were included. CSII was associated with improved glycosylated hemoglobin control in the first (MD, –0.34; 95% CI, –0.49 to –0.18) and second trimesters (MD, –0.15; 95% CI, –0.29 to –0.01), alongside reduced insulin requirements in early pregnancy (standardized mean difference, –0.43; 95% CI, –0.61 to –0.24). However, CSII was also linked to increased risks of cesarean delivery (RR, 1.11; 95% CI, 1.04-1.18), neonatal hypoglycemia (RR, 1.15; 95% CI, 1.03-1.30), and large-for-gestational-age infants (RR, 1.22; 95% CI, 1.11-1.34). No consistent differences were observed for preeclampsia, congenital malformations, or preterm birth. Heterogeneity across outcomes was moderate to high, reflecting variation in study design and quality.

**Conclusion:**

CSII offers measurable metabolic advantages during pregnancy by lowering glycosylated hemoglobin levels and reducing insulin needs. Nevertheless, these benefits do not consistently translate into improved maternal or neonatal outcomes and may be offset by higher obstetric and neonatal complication rates. Further well-powered randomized controlled trials are required to clarify the role of CSII and to balance metabolic gains against obstetric risks in managing pregnant women with T1DM.

## Graphical abstract

**Figure:**
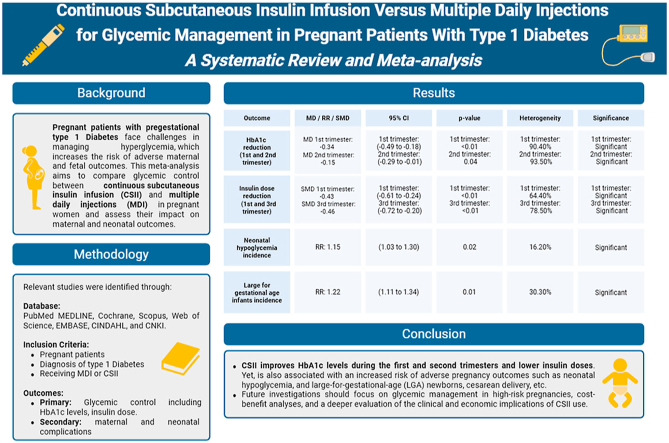

**Highlights**

• Continuous subcutaneous insulin infusion (CSII) lowers the hemoglobin A1C levels during the first and second trimesters
• CSII reduces insulin needs in early pregnancy
• CSII increases the risk of neonatal hypoglycemia
• CSII is linked to more large-for-gestational-age infants and cesarean deliveries
• Therapy choice should weigh metabolic gain versus obstetrical risk

**Clinical Relevance**

Continuous subcutaneous insulin infusion improves glycemic control in pregnant women with type 1 diabetes but may increase the risks of cesarean delivery and neonatal complications. Careful insulin therapy selection is needed to balance metabolic benefits and clinical outcomes.

## Introduction

Diabetes mellitus (DM) is recognized worldwide as one of the most prevalent health issues, affecting over 589 million people globally.^1^ Type 1 DM (T1DM) is characterized by T cell–mediated destruction of the insulin-producing pancreatic beta cells in the islets of Langerhans, eventually leading to complete insulin deficiency and hyperglycemia.^2^^,^^3^ The onset of T1DM typically occurs before the age of 30 years, and therefore, it can affect a significant number of individuals of reproductive age.^4^ Pregnant women with pregestational T1DM have an increased risk of adverse pregnancy outcomes, with studies showing that up to 30% to 40% experience complications such as preeclampsia, preterm birth, and neonatal hypoglycemia.^5^ Given the complexity of managing these high-risk pregnancies, individualized and careful treatment is essential to mitigate potential complications.

As in the general population, insulin therapy remains the standard approach for glycemic control in pregnant patients with T1DM,^6^ with 2 widely accepted administration methods: (1) multiple daily injections (MDIs) and (2) continuous subcutaneous insulin infusion (CSII).^7^ Emerging epidemiologic evidence suggests that CSII is associated with a reduction in chronic complications in both adult and pediatric populations, as well as with decreased cardiovascular mortality in adults with T1DM. Furthermore, CSII use has been consistently linked to lower hemoglobin A1C (HbA1C) levels.^7^

Despite these advancements, the ideal insulin delivery method for pregnant women with T1DM remains uncertain.^8^ A recent study suggests potential benefits of CSII over MDIs in terms of maternal and neonatal outcomes.^9^ This uncertainty raises important clinical questions regarding the safest and most effective insulin regimen for this population. Furthermore, the growing body of new evidence highlights the need to reassess and clarify current recommendations to reduce complications in pregnant women with T1DM.

This systematic review and meta-analysis (MA) aims to compare the effects of CSII and MDI on maternal and neonatal outcomes in pregnant women with T1DM. Given the limitations of previous analyses, an updated and methodologically robust review is needed to clarify the impact of insulin delivery methods on the maternal-fetal dyad and guide clinical decision making.

## Methods

The present systematic review followed the recommendations and criteria established by the Preferred Reporting Items for Systematic reviews and Meta-Analyses reporting guidelines. The protocol was registered at PROSPERO (CRD42025643129).

### Eligibility Criteria

We systematically reviewed studies published in English or Spanish, including randomized controlled trials (RCTs), cohort, and case-control studies. Eligible participants were pregnant women with T1DM. The intervention assessed was multiple daily insulin injections compared with CSII for glucose management. The primary outcomes included HbA1C, continuous glucose monitoring (CGM) metrics, or capillary glucose levels. The secondary outcomes included maternal and neonatal complications such as macrosomia, preterm birth, neonatal intensive care unit admission, hypoglycemia, respiratory distress, stillbirth, maternal hypoglycemia, ketoacidosis, and preeclampsia.

### Searching Methods

A systematic search was initially conducted on January 25, 2025, on PubMed, Cochrane, Scopus, Web of Science, Embase, CINAHL, and CNKI including terms related to type 1 diabetes (“Diabetes Mellitus, Type 1,” “T1DM”), pregnancy (“Pregnancy,” “pregnant women”), and insulin therapy methods (“continuous subcutaneous insulin infusion,” “CSII,” “insulin pump,” “multiple daily injections,” “MDI,”). All detailed search strategies are found in the supplementary material (Supplementary Tables 1-7).

### Selection of Studies

All references were exported to Rayyan, and duplicates were removed. Two authors independently completed the eligibility assessment, first by title and abstract analysis and, afterward, by full-text assessment. In disagreements between reviewers, consensus was reached with a third reviewer.

### Data Extraction

The data extraction was performed by 2 independent reviewers, and disagreements were solved by consensus; when multiple overlapping reports from the same study were identified, the information from the one containing the most relevant information or the first published report was included.

### Assessment of Risk of Bias

Two reviewers independently examined the methodological quality of the included studies using the Cochrane Risk of Bias 2 tool and Risk Of Bias In Non-randomized Studies-of Interventions for randomized and nonrandomized studies, respectively.^10^^,^^11^ Any disagreements were resolved by discussion with a third author.

### Statistical Analysis

An MA was performed using R version 3.4.3 (R Core Team). The pooled effect of the outcomes was examined using a random-effects MA (DerSimonian-Laird approach).^12^ Whenever the number of studies reporting an outcome of interest was insufficient, only a qualitative analysis of the results was performed. Effect sizes were expressed as relative risk (RR), mean difference (MD), standardized mean difference (SMD), and 95% confidence interval (CI). The I^2^ statistic assessed heterogeneity, and the following cutoff values used for interpretation: (1) <25%, (2) 25% to 50%, and (3) >50% were considered small, medium, and large heterogeneities, respectively.^13^ For all outcomes, sensitivity analyses according to the leave-one-out method were performed to determine the influence of individual studies on the overall effect.^14^ The Egger regression test examined publication bias when 10 or more reports with the same outcome were available.^15^ Whenever possible, subgroup analyses were performed for primary outcomes.

## Results

**Fig. 1:**
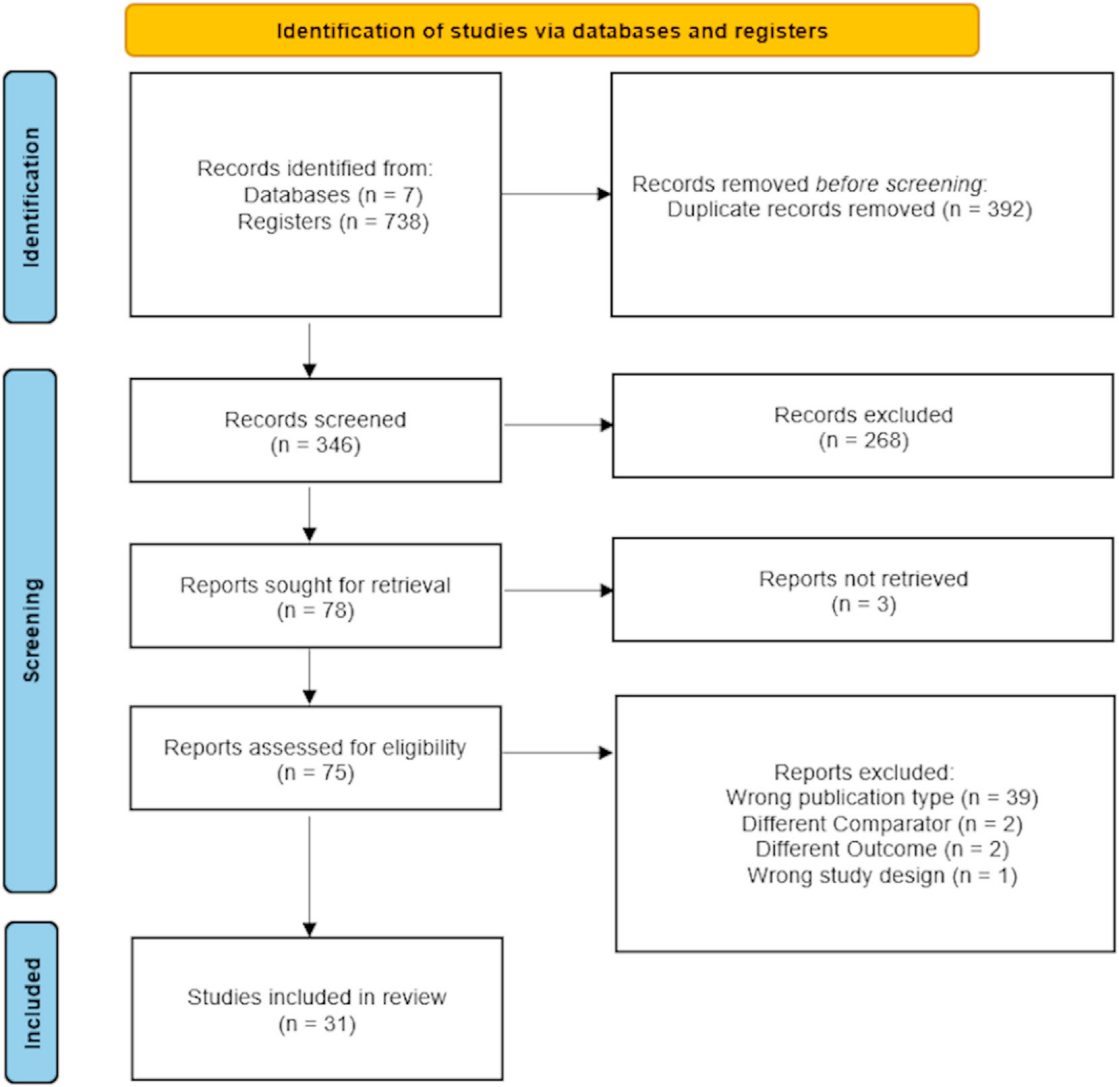
Preferred Reporting Items for Systematic reviews and Meta-Analyses (PRISMA) flow diagram. Flow diagram illustrating the study selection process for the systematic review. The initial search across 7 databases yielded a total of 738 records. After removing 392 duplicate records, 346 titles and abstracts were screened for relevance. Following the screening, 78 full-text reports were sought for detailed evaluation. Three of these could not be retrieved. Of the 75 reports assessed for eligibility, 44 were excluded based on specific exclusion criteria, including wrong publication type (*n* = 39), different comparator (*n* = 2), different outcome (*n* = 2), and wrong study design (*n* = 1). The remaining 31 studies met the inclusion criteria and were included in the systematic review. This process adheres to the PRISMA guidelines for reporting systematic reviews and meta-analyses.

In our initial search, we identified 738 potential articles. After removing 392 duplicate articles, 2 independent reviewers screened studies by title and abstract, followed by a full-text assessment to determine eligibility, leading to the exclusion of 268 articles. Three of the 78 articles sought for retrieval were not retrieved, leaving 75 articles for eligibility assessment. These remaining articles underwent screening for eligibility, excluding 39 due to inclusion criteria. The final selection included 31 studies. This process is summarized in our Preferred Reporting Items for Systematic reviews and Meta-Analyses flowchart (Fig. 1).

### Studies Characteristics

Thirty-one studies were included in this review, comprising a total of 6532 pregnant women. Of these, 24 were cohort studies involving 5747 patients, 5 were RCTs with 541 patients, and 2 were case-control studies including 244 patients. The studies were multinational in scope, with the majority conducted in Europe (64.5%, *n* = 20 studies), followed by North America (16.1%, *n* = 5 studies), Australia (6.5%, *n* = 2 studies), Asia (6.5%, *n* = 2 studies), South America (3.2%, *n* = 1 study), and multicenter studies accounting for 3.2% (*n* = 1 study). HbA1C management findings indicated that 64.5% (*n* = 20) of studies reported no significant difference between CSII and MDI, whereas 22.6% (*n* = 7) favored CSII, 3.2% (*n* = 1) favored MDI, and 3.2% (*n* = 1) did not compare HbA1C. Regarding maternal and neonatal outcomes, 74.2% (*n* = 23) of studies found no difference. However, 19.4% (*n* = 6) linked CSII to a higher incidence of pregnancy-related hypertension, ketoacidosis, cesarean delivery, large for gestational age (LGA), and neonatal hypoglycemia. On the contrary, CSII was associated with a lower risk of malformations. Additionally, 6.5 % (*n* = 2) did not directly compare outcomes. On the other hand, DM duration was significantly longer in the CSII group in 25.8% (*n* = 8) of studies. The aforementioned information is summarized in Table 1.^12^, ^13^, ^14^, ^15^, ^16^, ^17^, ^18^, ^19^, ^20^, ^21^, ^22^, ^23^, ^24^, ^25^, ^26^, ^27^, ^28^, ^29^, ^30^, ^31^, ^32^, ^33^, ^34^, ^35^, ^36^, ^37^, ^38^, ^39^, ^40^, ^41^, ^42^, ^43^, ^44^, ^45^, ^46^

**Table 1:** General Characteristics of the Included Studies

Author(s) and year	Country	Study design	Total age mean ± SD	Age mean ± SD (insulin pump)	Age mean ± SD (MDI)	Intervention type	Comparator type	Sample size insulin pump (*n*)	Sample size MDI (*n*)	Comorbidities	HbA1C preconception pump mean ± SD	Key points
Bruttomesso et al^16^ 2011	Italy	Cohort	31.8 ± 4.6	32.0 ± 4.4	31.4 ± 5.2	Insulin pump	MDIs	100	44	Retinopathy, nephropathy, HTN	7.20 ± 0.84	CSII users had earlier and better metabolic control than those with MDI; however, both had similar maternal/neonatal outcomes. There were no significant differences regarding severe hypoglycemia, diabetic ketoacidosis, or pregnancy complications. At parturition, patients using CSII had lower HbA1C levels and required less insulin.
Burkart et al^17^ 1988	Germany	RCT	28.45 ± 5.34	28.4 ± 5.3	28.49 ± 5.41	Insulin pump	MDIs	48	69	NA	NA	No difference in fetal/maternal outcomes. Severity of maternal diabetes significantly lower in CSII.
Coustan et al^18^ 1986	USA	RCT	28.5 ± 3.48	29 ± 3	28 ± 4	Insulin pump	MDIs	11	11	Retinopathy	8.6 ± 1.27	There were no differences between the 2 treatment groups in outpatient. Hypoglycemia did not increase in either treatment group.
Laatikainen et al^19^ 1987	Finland	RCT	26.82 ± 3.33	27 ± 3.5	26.7 ± 3.3	Insulin pump	MDIs	13	18	Retinopathy	NA	HbA1C levels decreased similarly in both the MDI and the CSII group. Dramatic progression was found in patients treated with CSII therapy compared with that in those treated with MDI. Within 8-12 weeks of the insertion of the insulin pump, macular edema and preproliferative changes in 2 patients
Mathiesen et al^20^ 2014	Denmark	RCT	NA	NA	NA	Insulin pump	MDIs	27	96	Retinopathy	NA	HbA1C third trimester CSII had lower results than MDI. Preterm birth and neonatal hypoglycemia rates were higher with MDI. LGA rate was higher with CSII. Key: Insulin pump therapy allows more freedom in eating patterns, which the patients may take advance of, and this may contribute to less optimal glycemic control.
Feig et al^21^ 2018	Multicenter	RCT	31.55 ± 4.6	31.9 ± 4.7	31.2 ± 4.5	Insulin pump	MDIs	125	123	Retinopathy, neuropathy, nephropathy, HTN	NA	MDI patients had better glycemic outcomes and greater HbA1C reduction and were more likely to reach target levels than CSII users. CSII patients had higher rates of neonatal hypoglycemia, gestational HTN, and NICU admissions.
Gabbe et al^22^ 2000	USA	Cohort	NA	NA	NA	Insulin pump	MDIs	36	24	Retinopathy, nephropathy, HTN, neuropathy	NA	No differences in HbA1C levels were observed among groups 1, 2, or 3 in the first, second, or third trimester. No significant differences in maternal or perinatal outcomes.
Kallas-Koeman et al^23^ 2014	Canada	Cohort	30.22 ± 4.99	31.5 ± 4.3	29.6 ± 5.2	Insulin pump	MDI	129	258	Retinopathy, nephropathy	7.05 ± 0.79	Patients using CSII were older and had a longer duration of diabetes and more retinopathy. The HbA1C level was lower in the first and third trimesters in CSII users, which persisted until the third trimester. Rates of diabetic ketoacidosis were similar in patients on insulin pumps. No increased incidence of hypoglycemia or more weight gain in pump users. More LGA in the pump group.
Neff et al^24^ 2014	Ireland	Cohort	34.69 ± 4.2	31.5 ± 5	35 ± 4	Insulin pump	MDIs	40	424	NA	6.5 ± 0.7	CSII significant in lower pregestational HbA1C levels; patients were older and have higher diabetes and cesarean delivery rates. No difference in fetal outcomes, just significant CSII for LGA and MDI for SGA.
Giménez et al^25^ 2007	Spain	Cohort	31.7 ± 3.63	32 ± 3.9	31.4 ± 3.4	Insulin pump	MDIs	29	29	NA	6.8 ± 1.2	No difference in HbA1C and maternal/neonatal outcomes.
Quirós et al^26^ 2014	Spain	Cohort	33.0 ± 5.0	NA	NA	Insulin pump	MDIs	16	20	NA	7.0 ± 1.3	No difference preconceptional HbA1C levels, difference in weight gain. CSII significant: higher HbA1C levels in third trimester and postpartum weight lost.
Lapolla et al^27^ 2003	Italy	Cohort	30.57 ± 4.78	31.6 ± 4.7	30.2 ± 4.8	Insulin pump	MDIs	25	68	NA	7.7 ± 1.6	CSII have HbA1C progressive reduction. No difference in maternal and neonatal outcomes. Suggest CSII in problematic, complicated cases.
Abell et al^28^ 2017	Australia	Cohort	29.7 ± 5.1	31 ± 4.6	29.3 ± 5.2	Insulin pump	MDIs	40	127	Retinopathy, microalbuminuria	7.87 ± 1.07	There were no significant differences in glycemic control and maternal/neonatal outcomes between CSII and MDI. CSII required lower insulin doses but did not provide better HbA1C levels or improved outcomes, so the study questions its routine use in pregnancy due to higher costs and complexity.
Wang et al^29^ 2022	USA	Cohort	31.0 ± 5.1	31.5 ± 4.4	30.2 ± 5.9	Insulin pump	MDIs	404	242	Chronic HTN, retinopathy, nephropathy	NA	CSII associated with better glycemic control as reflected by lower HbA1C values during the first and second trimesters. Use of insulin pump vs MDI therapy was associated with higher birth weights, birth weight percentiles, as well as increased odds of macrosomia and LGA. NICU admissions were more frequent in CSII users. No significant differences in preterm birth rates between CSII and MDI. Maternal outcomes reflected no major difference in cesarean delivery, hypoglycemia, DKA, or preeclampsia rates between groups.
Talaviya et al^30^ 2013	India	Cohort	30.67 ± 4.54	31.34 ± 5.11	30.2 ± 4.17	Insulin pump	MDIs	14	20	Retinopathy, nephropathy	8.72 ± 0.31	CSII gives better glycemic control and pregnancy outcome in pregnant women with type 1 diabetes than MDI treatment. CSII also decreases the daily insulin requirement compared with MDI. MDI showed higher hypoglycemia rate in newborn. No congenital abnormalities and no significant differences in cesarean delivery and preterm rates. Other secondary outcomes were not reported in this article.
Ogassavara et al^31^ 2023	Brazil	Cohort	26.69 ± 5.4	27.8 ± 5.7	26.4 ± 5.3	Insulin pump	MDIs	37	137	Chronic HTN, hypothyroidism, nephropathy, retinopathy	NA	Higher frequency of cesarean delivery and a lower occurrence of congenital malformations in the CSII group; however, the adjusted results may indicate that these associations are influenced by glycemic control. No major differences in glycemic control and other neonatal/maternal outcomes.
Chen et al^32^ 2007	Israel	Cohort	29.4 ± 4.9	29.6 ± 5.0	29.3 ± 4.9	Insulin pump	MDIs	30	60	Obesity	6.9 ± 0.6	Higher fetal hypoglycemia rates and maternal DKA rates in CSII than in MDI. No significant differences in glycemic control and other maternal/neonatal outcomes.
Sperling et al^33^ 2018	USA	Cohort	29.38 ± 5.67	30.2 ± 5.4	26.4 ± 5.8	Insulin pump	MDIs	49	107	Obesity	NA	No significant difference in HbA1C and maternal/fetal outcomes between CSII and MDI.
Kjölhede et al^34^ 2021	Sweden	Cohort	31 ± 4.7	31 ± 4	31 ± 5	Insulin pump	MDIs	54	131	Retinopathy, albuminuria	NA	Pregnant women with type 1 diabetes did not differ in glycemic control or maternal/neonatal outcomes, related to MDI or pump administration of insulin.
Cyganek et al^35^ 2010	Poland	Cohort	28.85 ± 4.32	Planned pregnancies: 29.7 ± 4.1	Planned pregnancies: 28.3 ± 4.4	Insulin pump	MDIs	38	58	Retinopathy, microalbuminuria	NA	Highlights the importance of pregnancy planning in patients with T1DM (better glycemic control and less complications). Both CSII and MDI provided similar glycemic control and maternal/fetal outcomes.
Jotic et al^36^ 2020	Serbia	Cohort	28.85 ± 5.30	27.6 ± 5.6	29.6 ± 5.0	Insulin pump	MDIs	48	80	NA	7.1 ± 0.1	Treatment with CSII resulted in a favorable reduction of HbA1C levels in the preconception period and each trimester in pregnancy. The incidence of LGA infants was lower in CSII. There were no significant differences in other neonatal outcomes between groups. The rate of preeclampsia was lower in the CSII group than in the MDI group and was the only significant difference in maternal outcomes.
Cypryk et al^37^ 2008	Poland	Cohort	27.86 ± 4.57	27.23 ± 4.78	28.08 ± 4.50	Insulin pump	MDIs	30	86	Retinopathy, nephropathy	NA	No significant difference in HbA1C and maternal/fetal outcomes between CSII and MDI.
Mantaj et al^38^ 2019	Poland	Cohort	27.96 ± 4,73	27.9 ± 4.5	28.0 ± 4.9	Insulin pump	MDIs	122	175	Nephropathy, retinopathy, chronic HTN	NA	The use of CSII in the treatment of pregnant women with T1DM was associated with a reduced number of neonatal complications presented as neonatal composite outcome but had no influence on maternal outcomes.
Kekäläinen et al^39^ 2016	Finland	Cohort	28.14 ± 5.27	31.3 ± 4.2	26.4 ± 5.0	Insulin pump	MDIs	48	87	Retinopathy, microalbuminuria, HTN	7.20 ± 0.86	CSII improved HbA1C earlier (especially in complicated cases); however, on the second trimester, both reached comparable glycemic control with no significant differences in pregnancy outcomes (maternal and neonatal).
Mello et al^40^ 2015	Italy	Cohort	32.85 ± 4.58	33.6 ± 3.6	31.4 ± 5.9	Insulin pump	MDIs	35	18	Retinopathy, nephropathy, neuropathy	6.73 ± 1.0	Both had similar maternal and neonatal outcomes and achieved similar glycemic control during pregnancy. MDI required higher dose on insulin on the third trimester.
Lason et al^41^ 2021	Poland	Cohort	NA	NA	29.1 ± 1.56	Insulin pump	MDIs	70	11	Retinopathy, albuminuria	NA	CSII with CGM resulted in better glycemic control and lower HbA1C. MDI patients had higher HbA1C levels but lower cesarean delivery rate, and overall pregnancy outcomes were similar between both groups.
Chico et al^42^ 2010	Spain	Cohort	NA	NA	NA	Insulin pump	MDIs	103	212	Retinopathy, nephropathy, chronic HTN		Groups differed in baseline and glycemic control HbA1C but not in maternal or fetal outcomes. CSII significant for lower insulin requirements.
Chico et al^43^ 2016	Spain	Cohort	NA	NA	NA	Insulin pump	MDIs	324	1210	Retinopathy, nephropathy, chronic HTN	6.33 ± 0.81	The type of basal insulin in pregnant women with T1DM was independently associated with metabolic variables and some fetal outcomes.
Dixon et al^44^ 2019	Australia	Cohort	29.7 ± 5	29.8 ± 4.94	29.7 ± 5.10	Insulin pump	MDIs	100	198	Retinopathy, neuropathy, nephropathy	NA	No difference in glycemic control between CSII and MDI. CSII is associated with an increased risk of LGA and preterm neonates. No other significant differences in maternal/neonatal outcomes.
Volpe et al^45^ 2010	Italy	Case-Control	30.37 ± 5	31.0+3.0	29.8 +6.3	Insulin pump	MDIs	20	22	Retinopathy and microalbuminuria, HTN	NA	Both CSII and MDI were equally effective controlling glucose, with similar HbA1C values and maternal/neonatal outcomes. CSII required less insulin than MDI. Both groups had high rates of LGA newborns and cesareans.
González-Romero et al^46^ 2010	Spain	Case-Control	29.55 ± 5.19	31.1 ± 4.0	29.23 ± 5.36	Insulin pump	MDIs	35	167	Retinopathy, nephropathy	6.62 ± 0.60%	CSII was significant for lower HbA1C levels before pregnancy and control during pregnancies also less insulin dose. No difference in maternal/neonatal outcomes.

Abbreviations: CSII = continuous subcutaneous insulin infusion; DKA = diabetic ketoacidosis; HbA1C = hemoglobin A1C; HTN = hypertension; LGA = large for gestational age; MDI = multiple daily injection; NA = not available; NICU = neonatal intensive care unit; RCT = randomized controlled trial; SD = standard deviation; SGA = small for gestational age; T1DM = type 1 diabetes mellitus.

### Risk of Bias Analysis

Among the 26 nonrandomized studies evaluated, 4 (15%) had a critical risk of bias, 9 studies (35%) had a serious risk, 9 studies (35%) presented some concerns, and 4 studies (15%) demonstrated a low risk of bias (Supplementary Fig. 1). For the 5 randomized studies, 1 study (20%) had a high risk of bias, whereas 4 studies (80%) demonstrated some concerns.

### MA Results

The MA for pregnancy glucose management, along with maternal-fetal outcomes, was performed with the included studies. A summary of the results is presented in Table 2.

**Table 2:** Meta-Analysis Outcomes

Outcome	Studies	Participants	Effect size (type)	95% CI	I^2^ (%)	*P* value
HbA1C in the first trimester	18	3408	MD, −0.34	−0.49 to −0.18	90.4	<.01
HbA1C in the second trimester	16	3312	MD, −0.15	−0.29 to −0.01	93.5	.04
HbA1C in the third trimester	22	3844	MD, −0.05	−0.17 to 0.06	82.3	.37
Preconceptional HbA1C	12	1513	MD, −0.32	−0.60 to −0.04	91.8	.02
Daily insulin dose (first trimester)	12	2720	SMD, −0.43	−0.61 to −0.24	64.4	<.01
Insulin dose (third trimester)	12	1353	SMD, −0.46	−0.72 to −0.20	78.5	<.01
Weight gain	16	4377	SMD, 0.05	−0.12 to 0.22	70.5	.58
Time with diabetes	23	4825	MD, 1.93	0.75 to 3.10	84.9	<.01
Cesarean delivery	23	5523	RR, 1.11	1.04 to 1.18	39	<.01
Preterm birth	21	5450	RR, 1.10	1.00 to 1.22	0	.05
Large for gestational age	22	5524	RR, 1.22	1.11 to 1.34	30.3	<.01
Small for gestational age	14	3908	RR, 1.06	0.62 to 1.81	0	.81
Congenital malformations	15	3870	RR, 0.91	0.59 to 1.41	26.5	.67
Preeclampsia and bleeding	14	3500	RR, 1.02	0.80 to 1.30	0	.86
Neonatal hypoglycemia	20	4902	RR, 1.15	1.03 to 1.30	16.2	.02
Neonatal jaundice	13	2264	RR, 1.14	0.85 to 1.28	30.1	.67
Birth weight	22	5212	MD, 32.17	−22.71 to 87.05	14.7	.25

Abbreviations: HbA1C = hemoglobin A1C; I^2^ = heterogeneity; MD = mean difference; RR = risk ratio; SMD = standardized mean difference.

All comparisons represent the effect of continuous subcutaneous insulin infusion (CSII) relative to multiple daily injection. For an RR of >1 or MD/SMD of >0, the outcome was higher in CSII; for an RR of <1 or MD/SMD of <0, the outcome was lower in CSII.

#### HbA1C in the First Trimester

**Fig. 2:**
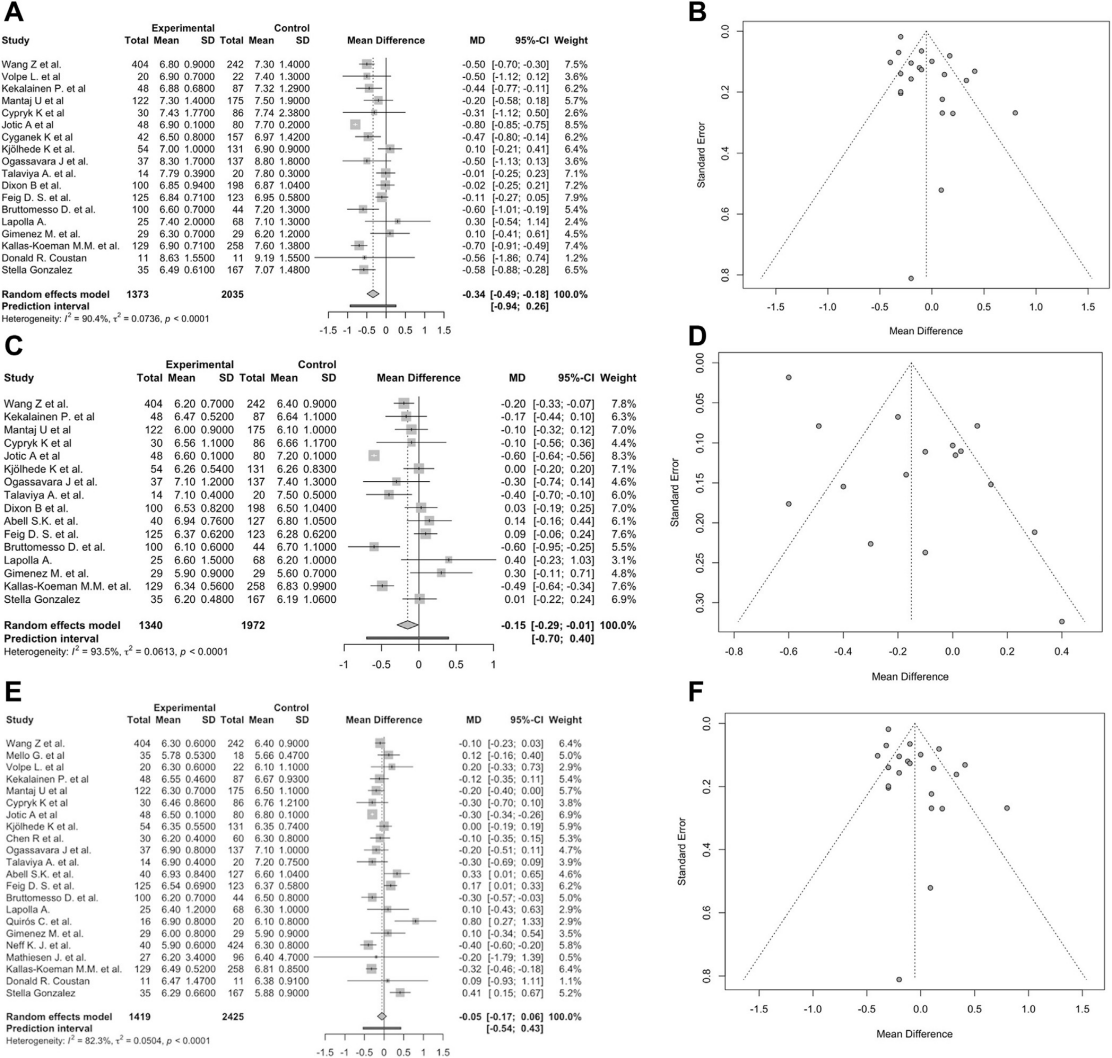
Hemoglobin A1C (HbA1C) levels in the first, second, and third trimesters. *A*, Forest plot detailing the mean difference (MD) and 95% CIs for the HbA1C levels in the first trimester. *B*, Funnel plot detailing publication bias for the HbA1C levels in the first trimester. *C*, Forest plot detailing the mean difference and 95% CIs for the HbA1C levels in the second trimester. *D*, Funnel plot detailing publication bias for the HbA1C levels in the second trimester. *E*, Forest plot detailing the mean difference and 95% CIs for the HbA1C levels in the third trimester. *F*, Funnel plot detailing publication bias for the HbA1C levels in the third trimester.

The MA included 18 studies with 3408 participants, with an MD of −0.34 (95% CI, −0.49 to −0.18; *P* < .01; I^2^ = 90.40%; Fig. 2 *A*). The funnel plot showed asymmetry, suggesting publication bias (Fig. 2 *B*). The Egger test confirmed small-study effects (*P* < .01). Subgroup analysis identified statistically significant differences based on the risk of bias (*P* = .04) and study design (*P* = .01; Supplementary Table 8). Studies with a critical risk of bias showed the largest effect (MD, −0.44; 95% CI, −0.59 to −0.28; I^2^ = 0.00%), whereas studies with a low risk of bias showed no consistent effect (MD, −0.56; 95% CI, −1.86 to 0.74). RCTs showed no significant differences (MD, −0.12; 95% CI, −0.28 to 0.04; I^2^ = 0.00%), whereas cohort studies showed a significant effect (MD, −0.33; 95% CI, −0.51 to −0.15; I^2^ = 90.20%). Sensitivity analysis did not identify any influential studies (Supplementary Table 9). Outlier analysis identified the study by Jotic et al^36^ as an outlier. Excluding this study reduced heterogeneity with an MD of −0.30 (95% CI, −0.44 to −0.15; *P* < .01; I^2^ = 70%), with an Egger test not significant (*P* = .95).

#### HbA1C in the Second Trimester

The MA included 16 studies with 3312 participants, with an MD of −0.15 (95% CI, −0.29 to −0.01; *P* = .04; I^2^ = 93.50%; Fig. 2 *C*). The funnel plot showed asymmetry, suggesting publication bias (Fig. 2 *D*). The Egger test confirmed small-study effects (*P* < .01). Subgroup analysis identified statistically significant differences based on the risk of bias (*P* = .04) and study design (*P* = .04; Supplementary Table 10). Studies with a critical risk of bias showed the largest effect (MD, −0.17; 95% CI, −0.28 to −0.07; I^2^ = 0.00%), whereas studies with a moderate risk of bias reported an MD of −0.15 (95% CI, −0.40 to 0.10; I^2^ = 81.6%). RCTs did show a nonsignificant effect (MD, 0.09; 95% CI, −0.06 to 0.24), whereas cohort studies showed a significant reduction in HbA1C levels (MD, −0.18; 95% CI, −0.34 to −0.03; I^2^ = 91.80%). Sensitivity analysis did not identify any influential studies (Supplementary Table 11). Outlier analysis identified the studies by Kallas-Koeman et al^23^ and Jotic et al^36^ as outliers. Excluding these studies reduced heterogeneity and resulted in an MD of −0.08 (95% CI, −0.19 to 0.03; *P* = .18; I^2^ = 59.20%), with an Egger test not significant (*P* = .97).

#### HbA1C in the Third Trimester

The MA included 22 studies with 3844 participants, with an MD of −0.05 (95% CI, −0.17 to 0.06; *P* = .37; I^2^ = 82.30%; Fig. 2 *E*). The funnel plot showed asymmetry, suggesting publication bias (Fig. 2 *F*). The Egger test confirmed small-study effects (*P* < .01). Subgroup analysis identified statistically significant differences based on the risk of bias (*P* = .01) and study design (*P* < .01; Supplementary Table 12). Studies with a critical risk of bias showed the largest reduction in HbA1C levels (MD, −0.13; 95% CI, −0.22 to −0.03; I^2^ = 0.00%), whereas studies with a low risk of bias showed no consistent effect (MD, 0.01; 95% CI, −0.85 to 0.86; I^2^ = 0.00%). Cohort studies reported an MD of −0.12 (95% CI, −0.23 to −0.01; I^2^ = 75.10%), whereas case-control studies and RCTs did not show a risk reduction. Sensitivity analysis did not identify any influential studies (Supplementary Table 13). Outlier analysis identified the studies by Kallas-Koeman et al,^23^ Neff et al,^24^ Quirós et al,^26^ Jotic et al,^36^ and González-Romero et al^46^ as outliers. Excluding these studies reduced heterogeneity and resulted in an MD of −0.05 (95% CI, −0.14 to 0.05; *P* = .32; I^2^ = 39.90%) and an Egger test not significant (*P* = .98).

#### Preeclampsia

The MA included 14 studies with 3500 participants, with an RR of 1.02 (95% CI, 0.80-1.30; *P* = .86; I^2^ = 0.00%; Supplementary Fig. 2). The funnel plot appeared symmetric (Supplementary Fig. 3), and the Egger test was not significant (*P* = .39). Subgroup and sensitivity analyses were not performed due to low heterogeneity.

#### Cesarean Delivery

The MA included 23 studies with 5523 participants, with an RR of 1.11 (95% CI, 1.04-1.18; *P* < .01; I^2^ = 39.00%; Supplementary Fig. 4). The funnel plot showed asymmetry, suggesting publication bias (Supplementary Fig. 5). The Egger test was not significant (*P* = .78). Subgroup analysis identified statistically significant differences based on the study design (*P* < .01; Supplementary Table 14). Cohort studies showed an increased incidence of cesarean delivery in the CSII group (RR, 1.12; 95% CI, 1.03-1.22; I^2^ = 47.60%). Sensitivity and outlier analyses identified the study by Neff et al^24^ as an influential study (Supplementary Table 15). Excluding this study reduced heterogeneity and resulted in an RR of 1.09 (95% CI, 1.03-1.15; *P* < .01; I^2^ = 17.20%). The corrected Egger test was not significant (*P* = .98).

#### Congenital Malformations

The MA included 15 studies with 3870 participants, with an RR of 0.91 (95% CI, 0.59-1.41; *P* = .67; I^2^ = 26.50%; Supplementary Fig. 6). The funnel plot showed asymmetry, suggesting publication bias (Supplementary Fig. 7). The Egger test was not significant (*P* = .15). Subgroup analysis identified statistically significant differences based on the time of pump initiation (*P* = .01; Supplementary Table 16), although this finding was based on a single study subgroup. Sensitivity and outlier analysis identified the studies by Burkart et al,^17^ Kallas-Koeman et al,^23^ and Chico et al^43^ as influential studies (Supplementary Table 17). Excluding these studies resulted in an RR of 0.88 (95% CI, 0.54-1.43; *P* = .57; I^2^ = 0.00%) and an Egger test not significant (*P* = .57).

#### Preterm Birth

The MA included 21 studies with 5450 participants, with an RR of 1.10 (95% CI, 1.00-1.22; *P* = .05; I^2^ = 0.00%; Supplementary Fig. 8). The funnel plot appeared symmetric (Supplementary Fig. 9). However, the Egger test indicated small-study effects (*P* = .01). Subgroup and sensitivity analyses were not performed due to low heterogeneity.

#### Large for Gestational Age

The MA included 22 studies with 5524 participants, with an RR of 1.22 (95% CI, 1.11-1.34; *P* < .01; I^2^ = 30.30%; Supplementary Fig. 10). The funnel plot showed asymmetry, suggesting potential publication bias (Supplementary Fig. 11). The Egger test was not significant (*P* = .89). Subgroup analysis identified statistically significant differences based on the risk of bias (*P* = .01; Supplementary Table 18). Studies with a critical risk of bias showed the highest RR (RR, 1.25; 95% CI, 1.17-1.33; I^2^ = 0.00%), whereas studies with a low risk of bias showed no consistent effect (RR, 0.67; 95% CI, 0.26-1.74; I^2^ = 0.00%). Sensitivity analysis did not identify any influential studies (Supplementary Table 19). Outlier analysis identified the study by Burkart et al^17^ as an outlier. Excluding this study reduced heterogeneity and resulted in an RR of 1.23 (95% CI, 1.14-1.33; *P* < .01; I^2^ = 18.60%) and an Egger test not significant (*P* = .98).

#### Small for Gestational Age

The MA included 14 studies with 3908 participants, with an RR of 1.06 (95% CI, 0.62-1.81; *P* = .81; I^2^ = 0.00%; Supplementary Fig. 12). The funnel plot showed asymmetry, suggesting potential publication bias (Supplementary Fig. 13). The Egger test did not indicate small-study effects (*P* = .21). Subgroup and sensitivity analyses were not performed due to low heterogeneity.

#### Neonatal Hypoglycemia

The MA included 20 studies with 4902 participants, with an RR of 1.15 (95% CI, 1.03-1.30; *P* = .02; I^2^ = 16.20%; Supplementary Fig. 14). The funnel plot showed asymmetry, suggesting potential publication bias (Supplementary Fig. 15). The Egger test did not indicate small-study effects (*P* = .47). Subgroup and sensitivity analyses were not performed due to low heterogeneity.

#### Neonatal Jaundice

The MA included 13 studies with 2264 participants, with an RR of 1.04 (95% CI, 0.85-1.28; *P* = .67; I^2^ = 30.10%) (Supplementary Fig. 16). The funnel plot appeared symmetric (Supplementary Fig. 17). The Egger test did not indicate small-study effects (*P* = .16). Subgroup analysis did not identify statistically significant differences (Supplementary Table 20). Sensitivity and outlier analyses did not identify any influential studies (Supplementary Table 21).

#### Birth Weight

The MA included 22 studies with 5212 participants, with an MD of 32.17 g (95% CI, −22.71 to 87.05; *P* = .25; I^2^ = 14.70%) (Supplementary Fig. 18). The funnel plot appeared symmetric (Supplementary Fig. 19). The Egger test did not indicate small-study effects (*P* = .44). Subgroup and sensitivity analyses were not performed due to low heterogeneity.

#### Daily Dose of Insulin in the First Trimester

**Fig. 3:**
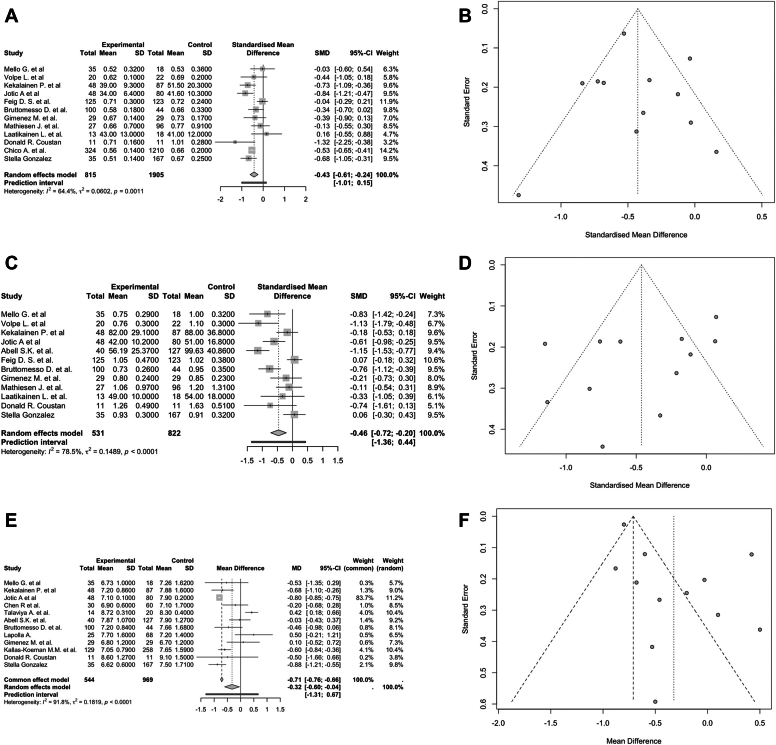
Insulin dose in First and Third Trimester and preconceptional hemoglobin A1C (HbA1C) levels. *A*, Forest plot detailing the mean difference and 95% CIs for insulin dose in the first trimester. *B*, Funnel plot detailing publication bias for insulin dose in the first trimester. *C*, Forest plot detailing the mean difference and 95% CIs for insulin dose in the third trimester. *D*, Funnel plot detailing publication bias for the insulin dose in the third trimester. *E*, Forest plot detailing the mean difference and 95% CIs for preconceptional HbA1C levels. *F*, Funnel plot detailing publication bias for preconceptional HbA1C levels. *SMD* = standardized mean difference.

The MA included 12 studies with 2720 participants, with an SMD of −0.43 (95% CI, −0.61 to −0.24; *P* < .01; I^2^ = 64.40%; Fig. 3 *A*). The funnel plot showed asymmetry, suggesting potential publication bias (Fig. 3 *B*). The Egger test did not indicate small-study effects (*P* = .71). Subgroup analysis identified statistically significant differences based on the risk of bias (*P* < .01; Supplementary Table 22). Studies with a critical risk of bias showed the largest effect (SMD, −0.73; 95% CI, −1.09 to −0.36), whereas studies with a low risk of bias showed no significant effect (SMD, −0.36; 95% CI, −1.15 to 0.43; I^2^ = 69.70%). Sensitivity and outlier analyses did not identify any influential studies (Supplementary Table 23).

#### Insulin Dose in the Third Trimester

The MA included 12 studies with 1353 participants, with an SMD of −0.46 (95% CI, −0.72 to −0.20; *P* < .01; I^2^ = 78.50%; Fig. 3 *C*). The funnel plot showed asymmetry, suggesting potential publication bias (Fig. 3 *D*). The Egger test did not indicate small-study effects (*P* = .15). Subgroup analysis identified statistically significant differences based on the risk of bias (*P* = .03) and study design (*P* = .035; Supplementary Table 24). Studies with a serious risk of bias showed a significant effect (SMD, −0.58; 95% CI, −1.09 to −0.08; I^2^ = 78.80%), whereas studies with a low risk of bias showed no consistent effect (SMD, −0.26; 95% CI, −0.59 to 0.08; I^2^ = 0.00%). Cohort studies showed a significant effect (SMD, −0.62; 95% CI, −0.93 to −0.32; I^2^ = 70.80%), whereas RCTs did not show a significant difference. Sensitivity analysis did not identify any influential studies (Supplementary Table 25). Outlier analysis identified the studies by Feig et al^21^ and Abell et al^28^ as outliers. Excluding these studies reduced heterogeneity and resulted in an SMD of −0.44 (95% CI, −0.68 to −0.20; *P* < .01; I^2^ = 60.00%) and an Egger test not significant (*P* = .29).

#### Weight Gain

The MA included 16 studies with 4377 participants, with an SMD of 0.05 (95% CI, −0.12 to 0.22; *P* = .58; I^2^ = 70.50%; Supplementary Fig. 20). The funnel plot showed asymmetry, suggesting potential publication bias (Supplementary Fig. 21). The Egger test did not indicate small-study effects (*P* = .66). Subgroup analysis did not identify statistically significant differences (Supplementary Table 26). Sensitivity analysis identified the study by Jotic et al^36^ as an influential study (Supplementary Table 27). Outlier analysis identified the studies by Jotic et al^36^ and González-Romero et al^46^ as outliers. Excluding these studies reduced heterogeneity (I^2^ = 0.00%) and resulted in an SMD of 0.13 (95% CI, 0.06-0.20; *P* < .01) and an Egger test not significant (*P* = .98).

#### Time With Diabetes

The MA included 23 studies with 4825 participants, with an MD of 1.93 years (95% CI, 0.75-3.10; *P* < .01; I^2^ = 84.90%; Supplementary Fig. 22). The funnel plot showed asymmetry, suggesting potential publication bias (Supplementary Fig. 23). The Egger test did not indicate small-study effects (*P* = .07). Subgroup analysis identified statistically significant differences based on the time of pump initiation (*P* = .04; Supplementary Table 28). Patients who started CSII before had an MD of 1.75 (95% CI, −0.29 to 3.79; I^2^ = 91.40%), whereas patients who started during pregnancy had an MD of 2.82 (95% CI, 1.41-4.22; I^2^ = 0.00%). Sensitivity analysis identified the study by Bruttomesso et al^16^ as an influential study (Supplementary Table 29). Outlier analysis identified the studies by Bruttomesso et al,^16^ Giménez et al,^25^ Abell et al,^28^ Kekäläinen et al,^39^ and Chico et al^42^ as outliers. Excluding these studies reduced heterogeneity and resulted in an MD of 2.49 years (95% CI, 1.53-3.45; *P* < .01; I^2^ = 57.80%). The corrected Egger test was not significant (*P* = .17).

#### Preconceptional HbA1C

The MA included 12 studies with 1513 participants, with an MD of −0.32 (95% CI, −0.60 to −0.04; *P* = .02; I^2^ = 91.80%; Fig. 3 *E*). The funnel plot showed asymmetry, suggesting potential publication bias (Fig. 3 *F*). The Egger test indicated small-study effects (*P* = .04). Subgroup analysis identified statistically significant differences based on the risk of bias (*P* = .05) and study design (*P* = .02; Supplementary Table 30). Studies with a serious risk of bias showed the largest effect (MD, −0.68; 95% CI, −0.97 to −0.39; I^2^ = 54.40%), whereas studies with a low risk of bias showed no significant effect (MD, −0.50; 95% CI, −1.66 to 0.66). Cohort studies showed a moderate effect (MD, −0.25; 95% CI, −0.55 to 0.05; I^2^ = 93.20%), whereas case-control studies showed the largest effect (MD, −0.88; 95% CI, −1.21 to −0.55). Sensitivity analysis identified the study by Talaviya et al^30^ as an influential study (Supplementary Table 31). Outlier analysis identified the studies by Jotic et al^36^ and Talaviya et al^30^ as outliers. Excluding these studies reduced heterogeneity (I^2^ = 63.30%) and resulted in an MD of −0.37 (95% CI, −0.63 to −0.11; *P* < .01). The corrected Egger test was not significant (*P* = .14).

## Discussion

The reviewed studies cover decades of insulin pump development, from early devices to modern systems with CGM and automated insulin delivery.^47^ Advanced hybrid closed loop (AHCL) systems show promise for improving glycemic control during pregnancy, offering better overnight stability and treatment satisfaction.^48^ However, many countries have not approved AHCL use in pregnancy, and access is limited in low- and middle-income regions. Consequently, MDIs and conventional pumps remain common in clinical practice. Studies using both old and new technologies were included to capture a broad evidence base, with heterogeneity managed through subgroup and sensitivity analyses.

In this manuscript, 31 studies and over 6000 participants provide comprehensive evidence on the effects of CSII compared with MDIs in pregnant women with T1DM. Results indicate that CSII is associated with a statistically significant reduction in HbA1C levels during the first and second trimesters and a decrease in insulin requirements for the first and third trimesters. Our analysis also shows a statistical association between CSII use and increased rates of cesarean deliveries, hypoglycemia, and LGA. Furthermore, CSII patients were diagnosed with T1DM for a longer period and had better achievement of glucose goals of preconceptional HbA1C, which may explain the better glycemic outcomes during the first and second trimesters.

Although HbA1C is widely used to assess long-term glycemic control, its reliability during pregnancy remains debated. Physiologic changes, such as increased red blood cell turnover and plasma volume expansion, can affect glycation rates and reduce HbA1C accuracy, particularly in the later stages of pregnancy.^49^ Despite these limitations, HbA1C remains a recommended marker by clinical guidelines and is frequently used in both research and practice, especially in the preconception period and first half of pregnancy, when its readings are more stable.^50^ In our MA, we observed significant reductions in HbA1C with CSII during the first and second trimesters. These findings may reflect better glycemic management before and early in pregnancy among CSII users.

Our qualitative synthesis and MA indicate the potential advantages of CSII during early pregnancy for improved blood glucose management and reduced insulin requirements. However, these benefits diminish in the third trimester. Our findings align with the work of Rys et al,^51^ who conducted a MA encompassing 47 studies with 7824 pregnancies in individuals with T1DM. They found that CSII yielded lower HbA1C levels in the first trimester, which decreased in subsequent trimesters but reduced daily insulin dose requirements persisted.^51^ Likewise, they noted increased gestational weight gain among CSII patients. Neonatal outcomes are also comparable to those in the study by Rys et al^51^ Both studies reported a higher prevalence of LGA newborns among CSII patients; however, our analysis revealed a higher RR. The authors also identified a reduced risk of small-for-gestational-age infants in the CSII cohort, contrasting with our MA, which did not detect a statistically significant difference in this outcome, likely due to stronger scientific rigor in study selection excluding abstracts, which we consider to be the main limitation of the study by Rys et al.^51^

Our study revealed an increased risk of cesarean delivery and neonatal hypoglycemia with CSII, contrasting a prior MA conducted by Ranasinghe et al^52^ in 2015, which found no significant differences in maternal and fetal outcomes in patients with T1DM and type 2 DM. Their inclusion of expectant mothers with type 2 DM highlights the need for studies such as ours targeting each diabetes type separately due to differing characteristics in pathophysiology and clinical management. Our study incorporated recent publications reflecting possible advancements in insulin pump technology alongside CGM systems. Furthermore, variability in study types exists; for instance, Rys et al^51^ combined RCTs and observational studies, including several conference abstracts, whereas our analysis focused primarily on fully published manuscripts, excluding any abstracts that may have contained incomplete information, high risk of bias, or an incorrect assessment of it, requiring cautious interpretation.

Our data indicated that pump users experienced improved prepregnancy glucose management, contributing to better glycemic management in early pregnancy. However, this advantage progressively diminished throughout gestation, aligning with MDI outcomes by the third trimester. Although these findings are statistically significant, they do not clinically demonstrate a difference in the advantages of CSII as an effective tool during pregnancy, especially considering the longer duration of T1DM onset in patients using CSII compared with MDIs, as observed in our MA. This may indicate a more prolonged exposure to metabolic changes and the complexities in disease management. In the first trimester, the superior preconception HbA1C levels in CSII users were still evident, as insulin resistance remained relatively mild. By the second trimester, increasing insulin resistance led to a convergence in glycemic management between the 2 groups, as the initial advantage of lower prepregnancy HbA1C became less distinguishable. By the third trimester, both groups exhibited comparable glycemic outcomes, likely due to the stricter metabolic management typically adopted during pregnancy and the full attenuation of preconception glycemic differences. Poor glycemic outcomes in the third trimester may help explain the observed associations with higher rates of LGA neonates, cesarean deliveries, and neonatal hypoglycemia in our analysis, patterns that are consistent with public research linking hyperglycemia in later pregnancy to fetal macrosomia and delivery complications.^53^^,^^54^ Although CSII was associated with improved HbA1C in early pregnancy, the increased risk of LGA observed in our analysis may seem contradictory. This can be partly explained by the fact that glycemic differences between CSII and MDI users diminished in the third trimester, as shown in our results. Additionally, patients using CSII had a longer duration of T1DM, which may reflect a more complex disease history and long-term metabolic burden. These factors may contribute to the observed association and highlight the importance of interpreting statistically significant findings within their broader clinical context. It is also acknowledged that adverse outcomes can arise from a history of poor and insufficient metabolic management during pregnancy.^4^^,^^22^^,^^55^^,^^56^ Considering this, we recommend an individualized approach that weighs the initial benefits and potential long-term implications of CSII versus MDIs, highlighting the importance of a supportive health care team and patient compliance. Furthermore, although our MA suggests a statistical correlation between CSII and certain adverse outcomes, these associations do not necessarily imply causality and should be interpreted in context, considering other clinical and economic factors such as cost/benefit ratios.

Several strengths are highlighted in this study. We collected data from a substantial sample size from diverse regions, representing the largest reported population for the examined variables, excluding abstracts. Our study also has important limitations. Despite systematic data extraction, potential human error or bias may have influenced the selection process. The MA revealed greater effect sizes in studies with a high risk of bias, demonstrating that some findings may overestimate the results. We acknowledge the asymmetry observed in our analysis that may represent a diminished quality of the studies included. Moreover, RCTs showed no significant difference in HbA1C reduction, whereas cohort studies did, highlighting the influence of observational study designs. Future research should consider follow-up differences, insulin dose management, and outcome standardization. Given that most current evidence is based on observational studies with a higher risk of bias, high-quality RCTs are needed. In addition, further research specifically evaluating modern CSII and AHCL systems in pregnancy is required to inform contemporary diabetes care and promote equitable access to advanced therapies globally.

## Conclusion

This MA comparing glucose management using CSII with MDIs in pregnant women with T1DM found that CSII significantly improved HbA1C levels during the first and second trimesters after adjusting for influencing factors. However, in terms of pregnancy outcomes, CSII use was associated with statistically significant increases in the risk of cesarean delivery, neonatal hypoglycemia, LGA newborns, and lower insulin doses. Future research is essential to refine perinatal HbA1C management. The primary limitation lies in the quality of the evidence rather than the sample size. Therefore, high-quality, well-designed studies are needed to address inconsistencies and strengthen conclusions. Future investigations should focus on glycemic management in high-risk pregnancies, cost-benefit analyses, and a deeper evaluation of the clinical and economic implications of CSII use.

## Disclosure

The authors have no conflicts of interest to disclose.
