# Supplementary material for: Continuous Subcutaneous Insulin Infusion Versus Multiple Daily Injections for Glycemic Management in Pregnant Women With Type 1 Diabetes: A Systematic Review and Meta-Analysis
Source: AACE Endocrinol Diabetes. 2025 Aug 21;12(4):314–27. doi: 10.1016/j.aed.2025.08.005 (PMC12744806; doi:10.1016/j.aed.2025.08.005)
Supplement: Supplementary Material [file mmc1.docx]

**SUPPLEMENTAL MATERIAL**

**Supplementary table 1.** Pubmed search

| SEARCH | RESULTS |
| --- | --- |
| ((Diabetes Mellitus, Type 1[Mesh]) OR (type 1 diabetes[Title/Abstract]) OR (type I diabetes[Title/Abstract]) OR (T1DM[Title/Abstract]) OR (type 1 diabetic[Title/Abstract]) OR (type I diabetic[Title/Abstract]) OR (type 1 diabetics[Title/Abstract]) OR (type I diabetics[Title/Abstract])) AND ((Pregnancy[Mesh]) OR (pregnant woman[Title/Abstract]) OR (pregnant women[Title/Abstract]) OR (pregnancy[Title/Abstract]) OR (pregnancies[Title/Abstract]) OR (expecting mother[Title/Abstract]) OR (expecting mothers[Title/Abstract]) OR (maternal[Title/Abstract])) AND  ((Insulin Infusion Systems[Mesh]) OR (continuous subcutaneous insulin infusion[Title/Abstract]) OR (CSII[Title/Abstract]) OR (insulin pump therapy[Title/Abstract]) OR (insulin pumps[Title/Abstract]) OR (insulin pump[Title/Abstract]) OR (insulin delivery systems[Title/Abstract]) OR (insulin delivery system[Title/Abstract]) OR (insulin infusion device[Title/Abstract]) OR (insulin infusion devices[Title/Abstract])) AND ((Injections, Subcutaneous[Mesh]) OR (multiple daily injection[Title/Abstract]) OR (multiple daily injections[Title/Abstract]) OR (MDI[Title/Abstract]) OR (daily insulin injections[Title/Abstract]) OR (daily insulin injection[Title/Abstract]) OR (subcutaneous injection[Title/Abstract]) OR (subcutaneous injections[Title/Abstract]) OR (insulin shots[Title/Abstract]) OR (insulin shot[Title/Abstract])) | 106 |

**Supplementary table 2.** Web of Science search

| SEARCH | RESULTS |
| --- | --- |
| ((ALL=("Diabetes Mellitus, Type 1") OR ALL=("type 1 diabetes") OR ALL=("type I diabetes") OR ALL=("T1DM") OR ALL=("type 1 diabetic") OR ALL=("type I diabetic") OR ALL=("type 1 diabetics") OR ALL=("type I diabetics"))) AND ((ALL=("Pregnancy") OR ALL=("pregnant woman") OR ALL=("pregnant women") OR ALL=("pregnancy") OR ALL=("pregnancies") OR ALL=("expecting mother") OR ALL=("expecting mothers") OR ALL=("maternal"))) AND ((ALL=("Insulin Infusion Systems") OR ALL=("continuous subcutaneous insulin infusion") OR ALL=("CSII") OR ALL=("insulin pump therapy") OR ALL=("insulin pumps") OR ALL=("insulin pump") OR ALL=("insulin delivery systems") OR ALL=("insulin delivery system") OR ALL=("insulin infusion device") OR ALL=("insulin infusion devices"))) AND ((ALL=("Injections, Subcutaneous") OR ALL=("multiple daily injection") OR ALL=("multiple daily injections") OR ALL=("MDI") OR ALL=("daily insulin injections") OR ALL=("daily insulin injection") OR ALL=("subcutaneous injection") OR ALL=("subcutaneous injections") OR ALL=("insulin shots") OR ALL=("insulin shot"))) | 127 |

**Supplementary table 3.** Embase search

| SEARCH | RESULTS |
| --- | --- |
| ('diabetes mellitus type 1'/exp OR 'type 1 diabetes':ti,ab OR 'type I diabetes':ti,ab OR 'T1DM':ti,ab OR 'type 1 diabetic':ti,ab OR 'type I diabetic':ti,ab OR 'type 1 diabetics':ti,ab OR 'type I diabetics':ti,ab) AND ('pregnancy'/exp OR 'pregnant woman':ti,ab OR 'pregnant women':ti,ab OR 'pregnancy':ti,ab OR 'pregnancies':ti,ab OR 'expecting mother':ti,ab OR 'expecting mothers':ti,ab OR 'maternal':ti,ab) AND ('insulin infusion system'/exp OR 'continuous subcutaneous insulin infusion':ti,ab OR 'CSII':ti,ab OR 'insulin pump therapy':ti,ab OR 'insulin pumps':ti,ab OR 'insulin pump':ti,ab OR 'insulin delivery system':ti,ab OR 'insulin delivery systems':ti,ab OR 'insulin infusion device':ti,ab OR 'insulin infusion devices':ti,ab) AND ('subcutaneous injection'/exp OR 'multiple daily injection':ti,ab OR 'multiple daily injections':ti,ab OR 'MDI':ti,ab OR 'daily insulin injections':ti,ab OR 'daily insulin injection':ti,ab OR 'subcutaneous injection':ti,ab OR 'subcutaneous injections':ti,ab OR 'insulin shots':ti,ab OR 'insulin shot':ti,ab) | 235 |

**Supplementary table 4.** CINAHL search

| SEARCH | RESULTS |
| --- | --- |
| ((MH "Diabetes Mellitus, Type 1" OR TX "type 1 diabetes" OR TX "type I diabetes" OR TX "T1DM" OR TX "type 1 diabetic" OR TX "type I diabetic" OR TX "type 1 diabetics" OR TX "type I diabetics")) AND ((MH "Pregnancy" OR TX "pregnant woman" OR TX "pregnant women" OR TX "pregnancy" OR TX "pregnancies" OR TX "expecting mother" OR TX "expecting mothers" OR TX "maternal")) AND ((MH "Insulin Infusion Systems" OR TX "continuous subcutaneous insulin infusion" OR TX "CSII" OR TX "insulin pump therapy" OR TX "insulin pumps" OR TX "insulin pump" OR TX "insulin delivery system" OR TX "insulin delivery systems" OR TX "insulin infusion device" OR TX "insulin infusion devices")) AND ((MH "Injections, Subcutaneous" OR TX "multiple daily injection" OR TX "multiple daily injections" OR TX "MDI" OR TX "daily insulin injections" OR TX "daily insulin injection" OR TX "subcutaneous injection" OR TX "subcutaneous injections" OR TX "insulin shots" OR TX "insulin shot")) | 33 |

**Supplementary table 5.** Scopus search

| SEARCH | RESULTS |
| --- | --- |
| (TITLE-ABS-KEY("Diabetes Mellitus, Type 1") OR TITLE-ABS-KEY("type 1 diabetes") OR TITLE-ABS-KEY("type I diabetes") OR TITLE-ABS-KEY("T1DM") OR TITLE-ABS-KEY("type 1 diabetic") OR TITLE-ABS-KEY("type I diabetic") OR TITLE-ABS-KEY("type 1 diabetics") OR TITLE-ABS-KEY("type I diabetics")) AND (TITLE-ABS-KEY("Pregnancy") OR TITLE-ABS-KEY("pregnant woman") OR TITLE-ABS-KEY("pregnant women") OR TITLE-ABS-KEY("pregnancy") OR TITLE-ABS-KEY("pregnancies") OR TITLE-ABS-KEY("expecting mother") OR TITLE-ABS-KEY("expecting mothers") OR TITLE-ABS-KEY("maternal")) AND (TITLE-ABS-KEY("Insulin Infusion Systems") OR TITLE-ABS-KEY("continuous subcutaneous insulin infusion") OR TITLE-ABS-KEY("CSII") OR TITLE-ABS-KEY("insulin pump therapy") OR TITLE-ABS-KEY("insulin pumps") OR TITLE-ABS-KEY("insulin pump") OR TITLE-ABS-KEY("insulin delivery system") OR TITLE-ABS-KEY("insulin delivery systems") OR TITLE-ABS-KEY("insulin infusion device") OR TITLE-ABS-KEY("insulin infusion devices")) AND (TITLE-ABS-KEY("Injections, Subcutaneous") OR TITLE-ABS-KEY("multiple daily injection") OR TITLE-ABS-KEY("multiple daily injections") OR TITLE-ABS-KEY("MDI") OR TITLE-ABS-KEY("daily insulin injections") OR TITLE-ABS-KEY("daily insulin injection") OR TITLE-ABS-KEY("subcutaneous injection") OR TITLE-ABS-KEY("subcutaneous injections") OR TITLE-ABS-KEY("insulin shots") OR TITLE-ABS-KEY("insulin shot")) | 129 |

**Supplementary table 6.** Cochrane search

| ID | SEARCH | RESULT |
| --- | --- | --- |
| 1 | [Diabetes Mellitus, Type 1] | 7,778 |
| 2 | ("type 1 diabetes":ti,ab OR "type I diabetes":ti,ab OR "T1DM":ti,ab OR "type 1 diabetic":ti,ab OR "type I diabetic":ti,ab OR "type 1 diabetics":ti,ab OR "type I diabetics":ti,ab) | 10,123 |
| 3 | [Pregnancy] | 34,889 |
| 4 | ("pregnant woman":ti,ab OR "pregnant women":ti,ab OR "pregnancy":ti,ab OR "pregnancies":ti,ab OR "expecting mother":ti,ab OR "expecting mothers":ti,ab OR "maternal":ti,ab) | 82,315 |
| 5 | [Insulin Infusion Systems] | 1,016 |
| 6 | ("continuous subcutaneous insulin infusion":ti,ab OR "CSII":ti,ab OR "insulin pump therapy":ti,ab OR "insulin pumps":ti,ab OR "insulin pump":ti,ab OR "insulin delivery system":ti,ab OR "insulin delivery systems":ti,ab OR "insulin infusion device":ti,ab OR "insulin infusion devices":ti,ab) | 2,350 |
| 7 | [Injections, Subcutaneous] | 5,800 |
| 8 | ("multiple daily injection":ti,ab OR "multiple daily injections":ti,ab OR "MDI":ti,ab OR "daily insulin injections":ti,ab OR "daily insulin injection":ti,ab OR "subcutaneous injection":ti,ab OR "subcutaneous injections":ti,ab OR "insulin shots":ti,ab OR "insulin shot":ti,ab) | 9,526 |
| 9 | #1 OR #2 | 12,498 |
| 10 | #3 OR #4 | 93,322 |
| 11 | #5 OR #6 | 2,752 |
| 12 | #7 OR #8 | 14,318 |
| 13 | #9 AND #10 AND #11 AND #12 | 25 |

**Supplementary table 7.** CNKI Search

| SEARCH | RESULTS |
| --- | --- |
| ((SU=("Diabetes Mellitus, Type 1" + "type 1 diabetes" + "type I diabetes" + "T1DM" + "type 1 diabetic" + "type I diabetic" + "type 1 diabetics" + "type I diabetics")) AND (SU=("Pregnancy" + "pregnant woman" + "pregnant women" + "pregnancy" + "pregnancies" + "expecting mother" + "expecting mothers" + "maternal")) AND (TKA=("Insulin Infusion Systems" + "continuous subcutaneous insulin infusion" + "CSII" + "insulin pump therapy" + "insulin pumps" + "insulin pump" + "insulin delivery system" + "insulin delivery systems" + "insulin infusion device" + "insulin infusion devices")) AND (TKA=("Injections, Subcutaneous" + "multiple daily injection" + "multiple daily injections" + "MDI" + "daily insulin injections" + "daily insulin injection" + "subcutaneous injection" + "subcutaneous injections" + "insulin shots" + "insulin shot"))) | 69 |

### **Supplementary table 8.** Subgroup analysis HbA1c in the First Trimester

| **Variable** | **Subgroup** | **k** | **MD (95% CI)** | **I² (%)** | **p-value (Between Groups)** |
| --- | --- | --- | --- | --- | --- |
| **Risk of Bias** | Critical | 3 | -0.44 [-0.59; -0.28] | 0.0 |  |
|  | Serious | 6 | -0.45 [-0.75; -0.16] | 87.0 |  |
|  | Moderate | 7 | -0.24 [-0.53; 0.04] | 81.2 |  |
|  | Some Concerns | 1 | -0.11 [-0.27; 0.05] | -- |  |
|  | Low | 1 | -0.56 [-1.86; 0.74] | -- |  |
|  | **p-value** |  |  |  | 0.047 |
| **Study Design** | Cohort | 14 | -0.33 [-0.51; -0.15] | 90.2 |  |
|  | Case-Control | 2 | -0.56 [-0.84; -0.29] | 0.0 |  |
|  | RCT | 2 | -0.12 [-0.28; 0.04] | 0.0 |  |
|  | **p-value** |  |  |  | 0.015 |
| **Time Started** | Before | 12 | -0.31 [-0.52; -0.11] | 93.5 |  |
|  | Both | 4 | -0.45 [-0.66; -0.24] | 0.0 |  |
|  | During | 2 | -0.23 [-0.59; 0.13] | 0.0 |  |
|  | **p-value** |  |  |  | 0.493 |

### **Supplementary table 9.** Sensitivity analysis HbA1c in the First Trimester

| **Author** | **Effect (95% CI: Lower; Upper)** | **I²** | **DFFITS** | **Cook's D** | **QE (del)** | **Is Influential** |
| --- | --- | --- | --- | --- | --- | --- |
| Omitting Wang Z et al. | -0.33 (-0.49; -0.16) | 0.9084 | -0.1697 | 0.0305 | 174.72 | No |
| Omitting Volpe L. et al | -0.33 (-0.49; -0.17) | 0.9092 | -0.0829 | 0.0070 | 176.27 | No |
| Omitting Kekalainen P. et al | -0.33 (-0.50; -0.17) | 0.9087 | -0.0930 | 0.0091 | 175.18 | No |
| Omitting Mantaj U et al | -0.35 (-0.51; -0.18) | 0.9066 | 0.0957 | 0.0095 | 171.37 | No |
| Omitting Cypryk K et al | -0.34 (-0.50; -0.18) | 0.9090 | 0.0045 | 0.00002 | 175.84 | No |
| Omitting Jotic A et al | -0.30 (-0.44; -0.15) | 0.7010 | -0.6164 | 0.2839 | 53.52 | No |
| Omitting Cyganek K et al | -0.33 (-0.49; -0.16) | 0.9088 | -0.1174 | 0.0145 | 175.53 | No |
| Omitting Kjölhede K et al. | -0.37 (-0.53; -0.22) | 0.8965 | 0.4152 | 0.1591 | 154.54 | No |
| Omitting Ogassavara J et al. | -0.33 (-0.49; -0.17) | 0.9092 | -0.0827 | 0.0070 | 176.27 | No |
| Omitting Talaviya A. et al. | -0.36 (-0.52; -0.21) | 0.8939 | 0.3311 | 0.1058 | 150.76 | No |
| Omitting Dixon B et al. | -0.36 (-0.52; -0.20) | 0.8931 | 0.3236 | 0.1015 | 149.67 | No |
| Omitting Feig D. S. et al. | -0.36 (-0.52; -0.19) | 0.8804 | 0.2398 | 0.0585 | 133.78 | No |
| Omitting Bruttomesso D. et al. | -0.32 (-0.49; -0.16) | 0.9093 | -0.1917 | 0.0375 | 176.42 | No |
| Omitting Lapolla A. | -0.36 (-0.51; -0.20) | 0.9068 | 0.2035 | 0.0412 | 171.76 | No |
| Omitting Gimenez M. et al. | -0.36 (-0.52; -0.20) | 0.9050 | 0.2662 | 0.0697 | 168.41 | No |
| Omitting Kallas-Koeman M.M. et al. | -0.31 (-0.47; -0.15) | 0.9091 | -0.3670 | 0.1285 | 175.96 | No |
| Omitting Donald R. Coustan | -0.34 (-0.49; -0.18) | 0.9093 | -0.0376 | 0.0014 | 176.42 | No |
| Omitting Stella Gonzalez | -0.32 (-0.49; -0.16) | 0.9093 | -0.2133 | 0.0468 | 176.33 | No |

### **Supplementary table 10.** Subgroup analysis HbA1c in the Second Trimester

| **Variable** | **Subgroup** | **k** | **MD (95% CI)** | **I² (%)** | **p-value (Between Groups)** |
| --- | --- | --- | --- | --- | --- |
| **Risk of Bias** | Critical | 3 | -0.17 [-0.28; -0.07] | 0.0 |  |
|  | Serious | 4 | -0.19 [-0.51; 0.13] | 95.2 |  |
|  | Moderate | 8 | -0.15 [-0.40; 0.10] | 81.6 |  |
|  | Some Concerns | 1 | 0.09 [-0.06; 0.24] | -- |  |
|  | **p-value** |  |  |  | 0.043 |
| **Study Design** | Cohort | 14 | -0.18 [-0.34; -0.03] | 91.8 |  |
|  | RCT | 1 | 0.09 [-0.06; 0.24] | -- |  |
|  | Case-Control | 1 | 0.01 [-0.22; 0.24] | -- |  |
|  | **p-value** |  |  |  | 0.043 |
| **Time Started** | Before | 12 | -0.14 [-0.32; 0.04] | 94.8 |  |
|  | Both | 3 | -0.18 [-0.39; 0.02] | 0.0 |  |
|  | During | 1 | -0.10 [-0.32; 0.12] | -- |  |
|  | **p-value** |  |  |  | 0.859 |

### **Supplementary table 11.** Sensitivity analysis HbA1c in the Second Trimester

| **Author** | **Effect (95% CI: Lower; Upper)** | **I²** | **DFFITS** | **Cook's D** | **QE (del)** | **Is Influential** |
| --- | --- | --- | --- | --- | --- | --- |
| Omitting Wang Z et al. | -0.15 (-0.30; 0.01) | 0.9347 | -0.0758 | 0.0063 | 214.39 | No |
| Omitting Kekalainen P. et al | -0.15 (-0.30; 0.00) | 0.9383 | -0.0359 | 0.0014 | 227.00 | No |
| Omitting Mantaj U et al | -0.15 (-0.30; -0.00) | 0.9364 | 0.0323 | 0.0011 | 220.24 | No |
| Omitting Cypryk K et al | -0.15 (-0.30; -0.01) | 0.9389 | 0.0201 | 0.0004 | 229.29 | No |
| Omitting Jotic A et al | -0.11 (-0.24; 0.01) | 0.7485 | -0.6475 | 0.2830 | 55.67 | No |
| Omitting Kjölhede K et al. | -0.16 (-0.31; -0.01) | 0.9334 | 0.1447 | 0.0220 | 210.21 | No |
| Omitting Ogassavara J et al. | -0.14 (-0.29; 0.00) | 0.9394 | -0.1109 | 0.0127 | 231.20 | No |
| Omitting Talaviya A. et al. | -0.13 (-0.28; 0.01) | 0.9395 | -0.2273 | 0.0528 | 231.56 | No |
| Omitting Dixon B et al. | -0.16 (-0.31; -0.02) | 0.9335 | 0.1767 | 0.0323 | 210.52 | No |
| Omitting Abell S.K. et al. | -0.17 (-0.31; -0.03) | 0.9350 | 0.2679 | 0.0708 | 215.31 | No |
| Omitting Feig D. S. et al. | -0.17 (-0.32; -0.03) | 0.9215 | 0.2797 | 0.0776 | 178.38 | No |
| Omitting Bruttomesso D. et al. | -0.13 (-0.27; 0.01) | 0.9395 | -0.3563 | 0.1206 | 231.28 | No |
| Omitting Lapolla A. | -0.17 (-0.31; -0.03) | 0.9376 | 0.2518 | 0.0624 | 224.48 | No |
| Omitting Gimenez M. et al. | -0.17 (-0.31; -0.04) | 0.9359 | 0.3388 | 0.1098 | 218.35 | No |
| Omitting Kallas-Koeman M.M. et al. | -0.12 (-0.27; 0.02) | 0.9396 | -0.3889 | 0.1410 | 231.75 | No |
| Omitting Stella Gonzalez | -0.16 (-0.31; -0.01) | 0.9345 | 0.1507 | 0.0237 | 213.86 | No |

### **Supplementary table 12.** Subgroup analysis HbA1c in the Third Trimester

| **Variable** | **Subgroup** | **k** | **MD (95% CI)** | **I² (%)** | **p-value (Between Groups)** |
| --- | --- | --- | --- | --- | --- |
| **Risk of Bias** | Critical | 3 | -0.13 [-0.22; -0.03] | 0.0 |  |
|  | Serious | 8 | 0.07 [-0.17; 0.30] | 89.2 |  |
|  | Moderate | 8 | -0.16 [-0.34; 0.02] | 65.7 |  |
|  | Some Concerns | 1 | 0.17 [ 0.01; 0.33] | -- |  |
|  | Low | 2 | 0.01 [-0.85; 0.86] | 0.0 |  |
|  | **p-value** |  |  |  | 0.017 |
| **Study Design** | Cohort | 17 | -0.12 [-0.23; -0.01] | 75.1 |  |
|  | Case-Control | 2 | 0.37 [ 0.14; 0.60] | 0.0 |  |
|  | RCT | 3 | 0.16 [ 0.01; 0.32] | 0.0 |  |
|  | **p-value** |  |  |  | 0.0001 |
| **Time Started** | Before | 17 | -0.01 [-0.16; 0.13] | 86.3 |  |
|  | Both | 3 | -0.18 [-0.35; -0.01] | 0.0 |  |
|  | During | 2 | -0.19 [-0.39; 0.01] | 0.0 |  |
|  | **p-value** |  |  |  | 0.237 |

### **Supplementary table 13.** Sensitivity analysis HbA1c in the Third Trimester

| **Author** | **Effect (95% CI: Lower; Upper)** | **I²** | **DFFITS** | **Cook's D** | **QE (del)** | **Is Influential** |
| --- | --- | --- | --- | --- | --- | --- |
| Omitting Wang Z et al. | -0.0486 (-0.1733; 0.0761) | 0.8248 | -0.0826 | 0.0075 | 114.1431 | No |
| Omitting Mello G. et al | -0.0622 (-0.1830; 0.0586) | 0.8219 | 0.1403 | 0.0202 | 112.3121 | No |
| Omitting Volpe L. et al | -0.0610 (-0.1795; 0.0576) | 0.8275 | 0.1212 | 0.0148 | 115.9192 | No |
| Omitting Kekalainen P. et al | -0.0483 (-0.1716; 0.0751) | 0.8299 | -0.0894 | 0.0085 | 117.5958 | No |
| Omitting Mantaj U et al | -0.0435 (-0.1663; 0.0793) | 0.8311 | -0.1674 | 0.0296 | 118.3875 | No |
| Omitting Cypryk K et al | -0.0432 (-0.1633; 0.0768) | 0.8310 | -0.1753 | 0.0313 | 118.3707 | No |
| Omitting Jotic A et al | -0.0356 (-0.1555; 0.0842) | 0.7299 | -0.3080 | 0.0934 | 74.0447 | No |
| Omitting Kjölhede K et al. | -0.0554 (-0.1790; 0.0683) | 0.8228 | 0.0263 | 0.0007 | 112.8532 | No |
| Omitting Chen R et al. | -0.0495 (-0.1728; 0.0738) | 0.8296 | -0.0697 | 0.0052 | 117.3794 | No |
| Omitting Ogassavara J et al. | -0.0453 (-0.1672; 0.0766) | 0.8311 | -0.1397 | 0.0203 | 118.4408 | No |
| Omitting Talaviya A. et al. | -0.0430 (-0.1631; 0.0770) | 0.8310 | -0.1791 | 0.0327 | 118.3646 | No |
| Omitting Abell S.K. et al. | -0.0742 (-0.1887; 0.0403) | 0.8119 | 0.3566 | 0.1188 | 106.3051 | No |
| Omitting Feig D. S. et al. | -0.0688 (-0.1882; 0.0505) | 0.7847 | 0.2562 | 0.0648 | 92.9039 | No |
| Omitting Bruttomesso D. et al. | -0.0402 (-0.1605; 0.0801) | 0.8309 | -0.2263 | 0.0521 | 118.2412 | No |
| Omitting Lapolla A. | -0.0577 (-0.1771; 0.0618) | 0.8290 | 0.0661 | 0.0044 | 116.9542 | No |
| Omitting Quirós C. et al. | -0.0820 (-0.1895; 0.0256) | 0.8070 | 0.4995 | 0.2266 | 103.6462 | No |
| Omitting Gimenez M. et al. | -0.0586 (-0.1787; 0.0614) | 0.8280 | 0.0818 | 0.0068 | 116.2693 | No |
| Omitting Neff K. J. et al. | -0.0340 (-0.1502; 0.0822) | 0.8272 | -0.3438 | 0.1103 | 115.7339 | No |
| Omitting Mathiesen J. et al. | -0.0528 (-0.1698; 0.0641) | 0.8312 | -0.0152 | 0.0002 | 118.4810 | No |
| Omitting Kallas-Koeman M.M. et al. | -0.0360 (-0.1553; 0.0833) | 0.8288 | -0.3022 | 0.0897 | 116.8197 | No |
| Omitting Donald R. Coustan | -0.0550 (-0.1726; 0.0625) | 0.8307 | 0.0221 | 0.0005 | 118.1009 | No |
| Omitting Stella Gonzalez | -0.0850 (-0.1929; 0.0228) | 0.7880 | 0.5838 | 0.2783 | 94.3404 | No |

### **Supplementary table 14.** Subgroup analysis Cesarean Delivery

| **Variable** | **Subgroup** | **k** | **RR (95% CI)** | **I² (%)** | **p-value (Between Groups)** |
| --- | --- | --- | --- | --- | --- |
| **Risk of Bias** | Critical | 3 | 1.17 [0.57; 2.40] | 43.8 |  |
|  | Serious | 8 | 1.08 [0.95; 1.23] | 47.7 |  |
|  | Moderate | 9 | 1.14 [1.00; 1.29] | 48.7 |  |
|  | Some Concerns | 1 | 1.08 [0.89; 1.31] | -- |  |
|  | Low | 2 | 1.11 [0.50; 2.45] | 0.0 |  |
|  | **p-value** |  |  |  | 0.964 |
| **Study Design** | Cohort | 18 | 1.12 [1.03; 1.22] | 47.6 |  |
|  | Case-Control | 2 | 0.99 [0.97; 1.02] | 0.0 |  |
|  | RCT | 3 | 1.08 [0.98; 1.20] | 0.0 |  |
|  | **p-value** |  |  |  | <0.0001 |
| **Time Started** | Before | 16 | 1.10 [1.02; 1.18] | 43.8 |  |
|  | Both | 5 | 1.13 [0.78; 1.64] | 51.8 |  |
|  | During | 2 | 1.11 [0.50; 2.45] | 0.0 |  |
|  | **p-value** |  |  |  | 0.973 |

### **Supplementary table 15.** Sensitivity analysis Cesarean Delivery

| **Author** | **Effect (95% CI: Lower; Upper)** | **I²** | **DFFITS** | **Cook's D** | **QE (del)** | **Is Influential** |
| --- | --- | --- | --- | --- | --- | --- |
| Omitting Wang Z et al. | 1.12 (1.04; 1.20) | 0.3508 | -0.3256 | 0.1076 | 32.35 | No |
| Omitting Lason I et al | 1.10 (1.04; 1.17) | 0.3726 | 0.0800 | 0.0064 | 33.47 | No |
| Omitting Volpe L. et al | 1.12 (1.05; 1.20) | 0.3687 | -0.3428 | 0.1158 | 33.26 | No |
| Omitting Kekalainen P. et al | 1.11 (1.03; 1.18) | 0.4167 | 0.0527 | 0.0028 | 36.00 | No |
| Omitting Cypryk K et al | 1.12 (1.05; 1.19) | 0.3523 | -0.2676 | 0.0691 | 32.42 | No |
| Omitting Jotic A et al | 1.10 (1.04; 1.18) | 0.4034 | 0.0805 | 0.0065 | 35.20 | No |
| Omitting Kjölhede K et al. | 1.11 (1.03; 1.18) | 0.4174 | 0.0230 | 0.0005 | 36.04 | No |
| Omitting Sperling J et al. | 1.11 (1.04; 1.19) | 0.4101 | -0.1079 | 0.0120 | 35.60 | No |
| Omitting Chen R et al. | 1.11 (1.04; 1.18) | 0.4160 | -0.0235 | 0.0006 | 35.96 | No |
| Omitting Ogassavara J et al. | 1.10 (1.03; 1.17) | 0.3643 | 0.3566 | 0.1205 | 33.03 | No |
| Omitting Talaviya A. et al. | 1.11 (1.04; 1.18) | 0.4168 | 0.0124 | 0.0002 | 36.01 | No |
| Omitting Dixon B et al. | 1.11 (1.03; 1.19) | 0.4160 | -0.0284 | 0.0009 | 35.96 | No |
| Omitting Abell S.K. et al. | 1.11 (1.04; 1.19) | 0.4065 | -0.1274 | 0.0165 | 35.38 | No |
| Omitting Feig D. S. et al. | 1.11 (1.04; 1.19) | 0.4157 | -0.0365 | 0.0014 | 35.94 | No |
| Omitting Bruttomesso D. et al. | 1.11 (1.04; 1.19) | 0.4144 | -0.0559 | 0.0032 | 35.86 | No |
| Omitting Lapolla A. | 1.12 (1.05; 1.19) | 0.3635 | -0.2598 | 0.0655 | 32.99 | No |
| Omitting Neff K. J. et al. | 1.09 (1.03; 1.15) | 0.1721 | 0.6469 | 0.2865 | 25.36 | **Yes** |
| Omitting Kallas-Koeman M.M. et al. | 1.11 (1.03; 1.18) | 0.4173 | 0.0353 | 0.0013 | 36.04 | No |
| Omitting Gabbe G. et al. | 1.11 (1.04; 1.18) | 0.4159 | -0.0255 | 0.0007 | 35.96 | No |
| Omitting Donald R. Coustan | 1.11 (1.04; 1.18) | 0.4156 | -0.0120 | 0.0001 | 35.94 | No |
| Omitting Chico A. et al. | 1.09 (1.02; 1.16) | 0.2856 | 0.5509 | 0.2620 | 29.39 | No |
| Omitting Burkart W. et al. | 1.11 (1.03; 1.18) | 0.4168 | 0.0465 | 0.0022 | 36.01 | No |
| Omitting Stella Gonzalez | 1.11 (1.04; 1.19) | 0.4113 | -0.0713 | 0.0052 | 35.67 | No |

### **Supplementary table 16.** Subgroup analysis Congenital Malformations

| **Variable** | **Subgroup** | **k** | **RR (95% CI)** | **I² (%)** | **p-value (Between Groups)** |
| --- | --- | --- | --- | --- | --- |
| **Risk of Bias** | Critical | 2 | 0.67 [0.01; 69.70] | 0.0 |  |
|  | Serious | 3 | 1.54 [0.15; 15.36] | 46.9 |  |
|  | Moderate | 8 | 1.05 [0.73; 1.51] | 0.0 |  |
|  | Some Concerns | 1 | 1.48 [0.25; 8.68] | -- |  |
|  | Low | 1 | 0.14 [0.04; 0.59] | -- |  |
|  | **p-value** |  |  |  | 0.053 |
| **Study Design** | Cohort | 12 | 1.15 [0.87; 1.52] | 0.0 |  |
|  | RCT | 2 | 0.43 [0.00; 1125031.81] | 75.4 |  |
|  | Case-Control | 1 | 0.73 [0.17; 3.11] | -- |  |
|  | **p-value** |  |  |  | 0.595 |
| **Time Started** | Before | 9 | 1.16 [0.97; 1.40] | 0.0 |  |
|  | Both | 5 | 0.86 [0.18; 4.20] | 48.1 |  |
|  | During | 1 | 0.14 [0.04; 0.59] | -- |  |
|  | **p-value** |  |  |  | 0.013 |

### **Supplementary table 17.** Sensitivity analysis Congenital Malformations

| **Author** | **Effect (95% CI: Lower; Upper)** | **I²** | **DFFITS** | **Cook's D** | **QE (del)** | **Is Influential** |
| --- | --- | --- | --- | --- | --- | --- |
| Omitting Lason I et al | 0.89 (0.56; 1.44) | 0.3167 | 0.1065 | 0.0118 | 19.03 | No |
| Omitting Kekalainen P. et al | 0.92 (0.58; 1.47) | 0.2945 | -0.0451 | 0.0021 | 18.43 | No |
| Omitting Cypryk K et al | 0.94 (0.67; 1.33) | 0.1306 | -0.1638 | 0.0232 | 14.95 | No |
| Omitting Ogassavara J et al. | 0.99 (0.66; 1.48) | 0.1951 | -0.4054 | 0.1566 | 16.15 | No |
| Omitting Dixon B et al. | 0.92 (0.56; 1.51) | 0.2934 | -0.0095 | 0.0001 | 18.40 | No |
| Omitting Abell S.K. et al. | 0.91 (0.57; 1.46) | 0.3087 | 0.0304 | 0.0010 | 18.80 | No |
| Omitting Feig D. S. et al. | 0.88 (0.55; 1.41) | 0.3126 | 0.1868 | 0.0363 | 18.91 | No |
| Omitting Bruttomesso D. et al. | 0.88 (0.56; 1.39) | 0.3007 | 0.1857 | 0.0350 | 18.59 | No |
| Omitting Lapolla A. | 0.90 (0.56; 1.44) | 0.3165 | 0.0994 | 0.0103 | 19.02 | No |
| Omitting Gimenez M. et al. | 0.92 (0.58; 1.45) | 0.2979 | -0.0151 | 0.0002 | 18.51 | No |
| Omitting Kallas-Koeman M.M. et al. | 0.84 (0.51; 1.38) | 0.2752 | 0.4302 | 0.2019 | 17.94 | **Yes** |
| Omitting Gabbe G. et al. | 0.92 (0.57; 1.48) | 0.3032 | -0.0021 | 0.000005 | 18.66 | No |
| Omitting Chico A. et al. | 0.84 (0.51; 1.39) | 0.2903 | 0.4006 | 0.1783 | 18.32 | **Yes** |
| Omitting Burkart W. et al. | 1.14 (0.89; 1.46) | 0.0000 | -1.2314 | 1.1641 | 10.97 | **Yes** |
| Omitting Stella Gonzalez | 0.91 (0.56; 1.47) | 0.3075 | 0.0271 | 0.0008 | 18.77 | No |

### **Supplementary table 18.** Subgroup analysis Large for Gestational Age

| **Variable** | **Subgroup** | **k** | **RR (95% CI)** | **I² (%)** | **p-value (Between Groups)** |
| --- | --- | --- | --- | --- | --- |
| **Risk of Bias** | Critical | 2 | 1.25 [1.17; 1.33] | 0.0 |  |
|  | Serious | 9 | 1.25 [1.07; 1.46] | 37.4 |  |
|  | Moderate | 7 | 1.32 [1.15; 1.51] | 0.0 |  |
|  | Some Concerns | 1 | 1.00 [0.80; 1.24] | -- |  |
|  | Low | 3 | 0.67 [0.26; 1.74] | 0.0 |  |
|  | **p-value** |  |  |  | 0.012 |
| **Study Design** | Cohort | 16 | 1.25 [1.16; 1.36] | 12.2 |  |
|  | Case-Control | 2 | 1.44 [0.14; 14.50] | 0.0 |  |
|  | RCT | 4 | 0.85 [0.50; 1.46] | 29.1 |  |
|  | **p-value** |  |  |  | 0.056 |
| **Time Started** | Before | 17 | 1.23 [1.14; 1.34] | 20.4 |  |
|  | Both | 3 | 1.01 [0.21; 4.81] | 50.1 |  |
|  | During | 2 | 0.63 [0.00; 2769.52] | 21.3 |  |
|  | **p-value** |  |  |  | 0.521 |

### **Supplementary table 19.** Sensitivity analysis Large for Gestational Age

| **Author** | **Effect (95% CI: Lower; Upper)** | **I²** | **DFFITS** | **Cook's D** | **QE (del)** | **Is Influential** |
| --- | --- | --- | --- | --- | --- | --- |
| Omitting Wang Z et al. | 1.22 (1.09; 1.35) | 0.3316 | 0.0681 | 0.0054 | 29.92 | No |
| Omitting Mello G. et al | 1.22 (1.10; 1.35) | 0.3356 | -0.0073 | 0.00005 | 30.10 | No |
| Omitting Volpe L. et al | 1.22 (1.10; 1.34) | 0.3102 | 0.0986 | 0.0097 | 28.99 | No |
| Omitting Kekalainen P. et al | 1.22 (1.10; 1.35) | 0.3360 | 0.0160 | 0.0003 | 30.12 | No |
| Omitting Cypryk K et al | 1.23 (1.13; 1.34) | 0.2392 | -0.1160 | 0.0132 | 26.29 | No |
| Omitting Jotic A et al | 1.20 (1.09; 1.32) | 0.2220 | 0.4560 | 0.1864 | 25.71 | No |
| Omitting Kjölhede K et al. | 1.22 (1.09; 1.35) | 0.3312 | 0.0910 | 0.0091 | 29.90 | No |
| Omitting Sperling J et al. | 1.21 (1.10; 1.35) | 0.3287 | 0.1046 | 0.0115 | 29.79 | No |
| Omitting Chen R et al. | 1.22 (1.10; 1.35) | 0.3322 | 0.0739 | 0.0057 | 29.95 | No |
| Omitting Ogassavara J et al. | 1.22 (1.10; 1.35) | 0.3300 | 0.0715 | 0.0052 | 29.85 | No |
| Omitting Dixon B et al. | 1.21 (1.09; 1.34) | 0.3161 | 0.1832 | 0.0359 | 29.24 | No |
| Omitting Abell S.K. et al. | 1.22 (1.10; 1.35) | 0.3338 | 0.0610 | 0.0040 | 30.02 | No |
| Omitting Feig D. S. et al. | 1.24 (1.13; 1.37) | 0.2576 | -0.4302 | 0.1673 | 26.94 | No |
| Omitting Bruttomesso D. et al. | 1.23 (1.11; 1.36) | 0.3170 | -0.1730 | 0.0305 | 29.28 | No |
| Omitting Lapolla A. | 1.22 (1.10; 1.36) | 0.3336 | -0.0598 | 0.0038 | 30.01 | No |
| Omitting Neff K. J. et al. | 1.21 (1.10; 1.33) | 0.2773 | 0.2358 | 0.0535 | 27.67 | No |
| Omitting Mathiesen J. et al. | 1.23 (1.12; 1.35) | 0.2766 | -0.2099 | 0.0426 | 27.65 | No |
| Omitting Kallas-Koeman M.M. et al. | 1.21 (1.09; 1.34) | 0.3012 | 0.2438 | 0.0610 | 28.62 | No |
| Omitting Donald R. Coustan | 1.22 (1.10; 1.35) | 0.3287 | 0.0242 | 0.0006 | 29.79 | No |
| Omitting Chico A. et al. | 1.24 (1.12; 1.38) | 0.2311 | -0.3776 | 0.1432 | 26.01 | No |
| Omitting Burkart W. et al. | 1.23 (1.14; 1.33) | 0.1856 | -0.1552 | 0.0228 | 24.56 | No |
| Omitting Stella Gonzalez | 1.22 (1.10; 1.35) | 0.3344 | 0.0476 | 0.0023 | 30.05 | No |

### **Supplementary table 20.** Subgroup analysis Neonatal Jaundice

| **Variable** | **Subgroup** | **k** | **RR (95% CI)** | **I² (%)** | **p-value (Between Groups)** |
| --- | --- | --- | --- | --- | --- |
| **Risk of Bias** | Serious | 5 | 0.95 [0.42; 2.17] | 60.1 |  |
|  | Critical | 1 | 0.95 [0.50; 1.80] | -- |  |
|  | Moderate | 6 | 1.12 [0.87; 1.43] | 19.6 |  |
|  | Some Concerns | 1 | 0.98 [0.65; 1.50] | -- |  |
|  | **p-value** |  |  |  | 0.891 |
| **Study Design** | Case-Control | 2 | 0.73 [0.00; 1088.65] | 55.5 |  |
|  | Cohort | 10 | 1.08 [0.86; 1.36] | 31.0 |  |
|  | RCT | 1 | 0.98 [0.65; 1.50] | -- |  |
|  | **p-value** |  |  |  | 0.759 |
| **Time Started** | Before | 11 | 1.00 [0.78; 1.29] | 37.0 |  |
|  | Both | 2 | 1.22 [0.19; 7.87] | 0.0 |  |
|  | **p-value** |  |  |  | 0.297 |
|  |  |  |  |  |  |

### **Supplementary table 21.** Sensitivity analysis Neonatal Jaundice

| **Author** | **Effect (95% CI: Lower; Upper)** | **I²** | **DFFITS** | **Cook's D** | **QE (del)** | **Is Influential** |
| --- | --- | --- | --- | --- | --- | --- |
| Omitting Volpe L. et al | 1.03 (0.82; 1.29) | 0.3536 | 0.1384 | 0.0197 | 17.02 | No |
| Omitting Kekalainen P. et al | 1.04 (0.83; 1.31) | 0.3520 | 0.0106 | 0.0001 | 16.98 | No |
| Omitting Jotic A et al | 1.10 (0.92; 1.31) | 0.1336 | -0.6400 | 0.3534 | 12.70 | No |
| Omitting Sperling J et al. | 1.01 (0.82; 1.26) | 0.3206 | 0.2764 | 0.0766 | 16.19 | No |
| Omitting Chen R et al. | 1.02 (0.83; 1.26) | 0.3064 | 0.2029 | 0.0404 | 15.86 | No |
| Omitting Ogassavara J et al. | 1.01 (0.80; 1.26) | 0.3123 | 0.3633 | 0.1340 | 15.99 | No |
| Omitting Dixon B et al. | 0.99 (0.79; 1.25) | 0.2305 | 0.5020 | 0.2393 | 14.29 | No |
| Omitting Abell S.K. et al. | 1.03 (0.81; 1.30) | 0.3592 | 0.1234 | 0.0176 | 17.17 | No |
| Omitting Feig D. S. et al. | 1.04 (0.82; 1.32) | 0.3494 | 0.0124 | 0.0002 | 16.91 | No |
| Omitting Bruttomesso D. et al. | 1.08 (0.88; 1.32) | 0.2520 | -0.3728 | 0.1319 | 14.71 | No |
| Omitting Lapolla A. | 1.03 (0.82; 1.30) | 0.3589 | 0.0750 | 0.0058 | 17.16 | No |
| Omitting Kallas-Koeman M.M. et al. | 1.06 (0.84; 1.34) | 0.3097 | -0.1483 | 0.0247 | 15.94 | No |
| Omitting Stella Gonzalez | 1.09 (0.91; 1.31) | 0.1862 | -0.4716 | 0.2055 | 13.52 | No |

### **Supplementary table 22.** Subgroup analysis Daily Dose of Insulin in the First Trimester

| **Variable** | **Subgroup** | **k** | **SMD (95% CI)** | **I² (%)** | **p-value (Between Groups)** |
| --- | --- | --- | --- | --- | --- |
| **Risk of Bias** | Serious | 5 | -0.55 [-0.69; -0.42] | 36.1 |  |
|  | Critical | 1 | -0.73 [-1.09; -0.36] | -- |  |
|  | Some Concerns | 1 | -0.04 [-0.29; 0.21] | -- |  |
|  | Moderate | 2 | -0.35 [-0.65; -0.06] | 0.0 |  |
|  | Low | 3 | -0.36 [-1.15; 0.43] | 69.7 |  |
|  | **p-value** |  |  |  | 0.004 |
| **Study Design** | Cohort | 6 | -0.53 [-0.63; -0.43] | 38.6 |  |
|  | Case-Control | 2 | -0.62 [-0.93; -0.30] | 0.0 |  |
|  | RCT | 4 | -0.20 [-0.62; 0.22] | 59.0 |  |
|  | **p-value** |  |  |  | 0.266 |
| **Time Started** | Before | 9 | -0.39 [-0.59; -0.20] | 64.5 |  |
|  | Both | 1 | -0.73 [-1.09; -0.36] | -- |  |
|  | During | 2 | -0.55 [-1.99; 0.90] | 83.6 |  |
|  | **p-value** |  |  |  | 0.281 |

### **Supplementary table 23.** Sensitivity analysis Daily Dose of Insulin in the First Trimester

| **Author** | **Effect (95% CI: Lower; Upper)** | **I²** | **DFFITS** | **Cook's D** | **QE (del)** | **Is Influential** |
| --- | --- | --- | --- | --- | --- | --- |
| Omitting Mello G. et al | -0.45 (-0.64; -0.26) | 0.6448 | 0.2800 | 0.0779 | 28.72 | No |
| Omitting Volpe L. et al | -0.42 (-0.62; -0.22) | 0.6703 | -0.0081 | 0.00007 | 30.91 | No |
| Omitting Kekalainen P. et al | -0.39 (-0.58; -0.20) | 0.6444 | -0.3355 | 0.1119 | 28.65 | No |
| Omitting Jotic A et al | -0.38 (-0.56; -0.20) | 0.6164 | -0.4788 | 0.2020 | 26.57 | No |
| Omitting Feig D. S. et al. | -0.49 (-0.65; -0.32) | 0.4457 | 0.8511 | 0.4251 | 18.52 | No |
| Omitting Bruttomesso D. et al. | -0.43 (-0.64; -0.23) | 0.6657 | 0.0899 | 0.0091 | 30.49 | No |
| Omitting Gimenez M. et al. | -0.43 (-0.63; -0.22) | 0.6696 | 0.0299 | 0.0010 | 30.84 | No |
| Omitting Mathiesen J. et al. | -0.45 (-0.64; -0.26) | 0.6426 | 0.2888 | 0.0842 | 28.54 | No |
| Omitting Laatikainen L. et al. | -0.45 (-0.64; -0.26) | 0.6363 | 0.3021 | 0.0893 | 28.02 | No |
| Omitting Donald R. Coustan | -0.40 (-0.58; -0.21) | 0.6349 | -0.2963 | 0.0861 | 27.61 | No |
| Omitting Chico A. et al. | -0.41 (-0.62; -0.19) | 0.6359 | -0.1524 | 0.0284 | 28.15 | No |
| Omitting Stella Gonzalez | -0.40 (-0.59; -0.20) | 0.6533 | -0.2764 | 0.0790 | 29.40 | No |

**Supplementary table 24.** Subgroup analysis Insulin Dose in the Third Trimester

| **Variable** | **Subgroup** | **k** | **SMD (95% CI)** | **I² (%)** | **p-value (Between Groups)** |
| --- | --- | --- | --- | --- | --- |
| **Risk of Bias** | Serious | 4 | -0.58 [-1.09; -0.08] | 78.8 |  |
|  | Critical | 1 | -0.18 [-0.53; 0.18] | -- |  |
|  | Moderate | 3 | -0.73 [-1.24; -0.22] | 76.0 |  |
|  | Some Concerns | 1 | 0.07 [-0.18; 0.32] | -- |  |
|  | Low | 3 | -0.26 [-0.59; 0.08] | 0.0 |  |
|  | **p-value** |  |  |  | 0.027 |
| **Study Design** | Cohort | 6 | -0.62 [-0.93; -0.32] | 70.8 |  |
|  | Case-Control | 2 | -0.50 [-1.67; 0.67] | 89.8 |  |
|  | RCT | 4 | -0.09 [-0.35; 0.17] | 24.7 |  |
|  | **p-value** |  |  |  | 0.035 |
| **Time Started** | Before | 9 | -0.49 [-0.81; -0.17] | 83.7 |  |
|  | Both | 1 | -0.18 [-0.53; 0.18] | -- |  |
|  | During | 2 | -0.50 [-1.05; 0.06] | 0.0 |  |
|  | **p-value** |  |  |  | 0.377 |

**Supplementary table 25.** Sensitivity analysis Insulin Dose in the Third Trimester

| **Author** | **Effect (95% CI: Lower; Upper)** | **I²** | **DFFITS** | **Cook's D** | **QE (del)** | **Is Influential** |
| --- | --- | --- | --- | --- | --- | --- |
| Omitting Mello G. et al | -0.43 (-0.70; -0.16) | 0.7912 | -0.2133 | 0.0464 | 48.67 | No |
| Omitting Volpe L. et al | -0.41 (-0.66; -0.15) | 0.7783 | -0.3850 | 0.1403 | 45.72 | No |
| Omitting Kekalainen P. et al | -0.49 (-0.76; -0.21) | 0.7961 | 0.2351 | 0.0589 | 49.87 | No |
| Omitting Jotic A et al | -0.44 (-0.72; -0.16) | 0.7932 | -0.0957 | 0.0100 | 49.22 | No |
| Omitting Abell S.K. et al. | -0.37 (-0.61; -0.14) | 0.6870 | -0.7182 | 0.3871 | 32.73 | No |
| Omitting Feig D. S. et al. | -0.52 (-0.77; -0.26) | 0.7161 | 0.4938 | 0.2142 | 35.83 | No |
| Omitting Bruttomesso D. et al. | -0.43 (-0.70; -0.15) | 0.7797 | -0.2204 | 0.0507 | 46.24 | No |
| Omitting Gimenez M. et al. | -0.48 (-0.76; -0.20) | 0.7997 | 0.1739 | 0.0321 | 50.79 | No |
| Omitting Mathiesen J. et al. | -0.49 (-0.76; -0.22) | 0.7952 | 0.2612 | 0.0710 | 49.67 | No |
| Omitting Laatikainen L. et al. | -0.47 (-0.74; -0.19) | 0.8011 | 0.0799 | 0.0067 | 51.14 | No |
| Omitting Donald R. Coustan | -0.44 (-0.71; -0.17) | 0.7986 | -0.1042 | 0.0111 | 50.43 | No |
| Omitting Stella Gonzalez | -0.51 (-0.77; -0.25) | 0.7748 | 0.4193 | 0.1653 | 45.16 | No |

**Supplementary table 26.** Subgroup analysis Weight Gain

| **Variable** | **Subgroup** | **k** | **SMD (95% CI)** | **I² (%)** | **p-value (Between Groups)** |
| --- | --- | --- | --- | --- | --- |
| **Risk of Bias** | Critical | 4 | 0.16 [ 0.04; 0.28] | 0.0 |  |
|  | Serious | 6 | -0.06 [-0.51; 0.38] | 87.7 |  |
|  | Moderate | 4 | 0.13 [-0.02; 0.29] | 8.2 |  |
|  | Some Concerns | 1 | 0.00 [-0.25; 0.25] | -- |  |
|  | Low | 1 | -0.19 [-0.62; 0.24] | -- |  |
|  | **p-value** |  |  |  | 0.409 |
| **Study Design** | Cohort | 12 | 0.10 [-0.10; 0.29] | 70.2 |  |
|  | Case-Control | 2 | -0.09 [-1.01; 0.83] | 85.1 |  |
|  | RCT | 2 | -0.05 [-0.26; 0.17] | 0.0 |  |
|  | **p-value** |  |  |  | 0.596 |
| **Time Started** | Before | 12 | 0.01 [-0.21; 0.23] | 77.6 |  |
|  | Both | 3 | 0.18 [-0.05; 0.42] | 0.0 |  |
|  | During | 1 | 0.08 [-0.15; 0.31] | -- |  |
|  | **p-value** |  |  |  | 0.579 |

**Supplementary table 27.** Sensitivity analysis Weight Gain

| **Author** | **Effect (95% CI: Lower; Upper)** | **I²** | **DFFITS** | **Cook's D** | **QE (del)** | **Is Influential** |
| --- | --- | --- | --- | --- | --- | --- |
| Omitting Wang Z et al. | 0.04 (-0.15; 0.22) | 0.7111 | 0.1116 | 0.0138 | 49.06 | No |
| Omitting Lason I et al | 0.03 (-0.14; 0.20) | 0.7150 | 0.1795 | 0.0324 | 49.70 | No |
| Omitting Mello G. et al | 0.04 (-0.14; 0.21) | 0.7203 | 0.0886 | 0.0081 | 50.64 | No |
| Omitting Volpe L. et al | 0.03 (-0.14; 0.20) | 0.7152 | 0.1807 | 0.0329 | 49.70 | No |
| Omitting Kekalainen P. et al | 0.04 (-0.14; 0.22) | 0.7187 | 0.1124 | 0.0135 | 50.37 | No |
| Omitting Mantaj U et al | 0.04 (-0.14; 0.23) | 0.7217 | 0.0176 | 0.0003 | 50.91 | No |
| Omitting Jotic A et al | 0.10 (0.04; 0.17) | 0.3792 | -1.2160 | 0.4500 | 22.83 | **Yes** |
| Omitting Chen R et al. | 0.02 (-0.15; 0.19) | 0.7055 | 0.2751 | 0.0742 | 48.09 | No |
| Omitting Ogassavara J et al. | 0.05 (-0.14; 0.23) | 0.7217 | 0.0154 | 0.0003 | 50.91 | No |
| Omitting Feig D. S. et al. | 0.05 (-0.13; 0.23) | 0.7197 | -0.0522 | 0.0030 | 50.56 | No |
| Omitting Bruttomesso D. et al. | 0.05 (-0.13; 0.24) | 0.7184 | -0.0938 | 0.0095 | 50.32 | No |
| Omitting Lapolla A. | 0.02 (-0.15; 0.19) | 0.7056 | 0.2761 | 0.0746 | 48.11 | No |
| Omitting Mathiesen J. et al. | 0.06 (-0.12; 0.24) | 0.7134 | -0.1676 | 0.0292 | 49.43 | No |
| Omitting Kallas-Koeman M.M. et al. | 0.04 (-0.14; 0.22) | 0.7182 | 0.0877 | 0.0085 | 50.28 | No |
| Omitting Chico A. et al. | 0.04 (-0.14; 0.23) | 0.7194 | 0.0458 | 0.0024 | 50.50 | No |
| Omitting Stella Gonzalez | 0.08 (-0.08; 0.24) | 0.6475 | -0.4594 | 0.1777 | 40.25 | No |

### **Supplementary table 28.** Subgroup analysis Time with Diabetes

| **Variable** | **Subgroup** | **k** | **MD (95% CI)** | **I² (%)** | **p-value (Between Groups)** |
| --- | --- | --- | --- | --- | --- |
| **Risk of Bias** | Critical | 3 | 4.1916 [2.4699; 5.9134] | 40.6 |  |
|  | Serious | 10 | 1.5287 [-0.4782; 3.5357] | 86.6 |  |
|  | Moderate | 8 | 2.9793 [-0.4604; 6.4189] | 92.3 |  |
|  | Some Concerns | 1 | 0.5300 [-1.7585; 2.8185] | — |  |
|  | Low | 2 | 2.0698 [-0.1641; 4.3036] | 0.0 |  |
|  | **p-value** |  |  |  | 0.1057 |
| **Study Design** | Cohort | 19 | 2.3920 [0.6259; 4.1581] | 91.3 |  |
|  | Case-Control | 2 | 4.5023 [2.0169; 6.9877] | 0.0 |  |
|  | RCT | 3 | 1.3185 [-0.2800; 2.9170] | 0.0 |  |
|  | **p-value** |  |  |  | 0.1067 |
| **Time Started** | Both | 5 | 4.4780 [3.3123; 5.6437] | 0.0 |  |
|  | Before | 16 | 1.7480 [-0.2921; 3.7880] | 91.4 |  |
|  | During | 3 | 2.8160 [1.4148; 4.2171] | 0.0 |  |
|  | **p-value** |  |  |  | 0.0398 |

### **Supplementary table 29.** Sensitivity analysis Time with Diabetes

| **Author** | **Effect (95% CI: Lower; Upper)** | **I²** | **DFFITS** | **Cook's D** | **QE (del)** | **Is Influential** |
| --- | --- | --- | --- | --- | --- | --- |
| Omitting Lason I et al | 1.8519 (0.6345; 3.0692) | 0.8530 | 0.0664 | 0.0046 | 211.9763 | No |
| Omitting Mello G. et al | 1.9276 (0.7147; 3.1404) | 0.8560 | -0.0228 | 0.0005 | 214.0687 | No |
| Omitting Volpe L. et al | 1.8687 (0.6664; 3.0709) | 0.8549 | 0.0650 | 0.0043 | 213.2510 | No |
| Omitting Kekalainen P. et al | 1.7186 (0.5587; 2.8785) | 0.8409 | 0.2341 | 0.0542 | 202.1953 | No |
| Omitting Mantaj U et al | 1.8558 (0.6304; 3.0812) | 0.8507 | 0.0545 | 0.0031 | 210.6098 | No |
| Omitting Cypryk K et al | 1.7868 (0.5938; 2.9798) | 0.8492 | 0.1519 | 0.0235 | 208.7139 | No |
| Omitting Jotic A et al | 2.0950 (0.9128; 3.2772) | 0.8514 | -0.2540 | 0.0633 | 208.3664 | No |
| Omitting Cyganek K et al | 1.8791 (0.6560; 3.1022) | 0.8543 | 0.0324 | 0.0011 | 213.0244 | No |
| Omitting Kjölhede K et al. | 1.9286 (0.7331; 3.1241) | 0.8560 | -0.0164 | 0.0003 | 214.0880 | No |
| Omitting Sperling J et al. | 1.8795 (0.6567; 3.1022) | 0.8544 | 0.0322 | 0.0011 | 213.0576 | No |
| Omitting Chen R et al. | 2.0819 (0.8864; 3.2774) | 0.8513 | -0.2273 | 0.0515 | 207.8282 | No |
| Omitting Ogassavara J et al. | 1.7891 (0.5905; 2.9877) | 0.8474 | 0.1406 | 0.0203 | 207.4886 | No |
| Omitting Talaviya A. et al. | 2.0212 (0.8016; 3.2408) | 0.8550 | -0.1464 | 0.0220 | 212.1465 | No |
| Omitting Dixon B et al. | 1.9129 (0.6836; 3.1422) | 0.8553 | -0.0108 | 0.0001 | 213.7961 | No |
| Omitting Abell S.K. et al. | 2.1105 (0.9430; 3.2780) | 0.8507 | -0.2863 | 0.0791 | 207.8345 | No |
| Omitting Feig D. S. et al. | 2.0007 (0.7772; 3.2242) | 0.8557 | -0.1206 | 0.0150 | 213.1297 | No |
| Omitting Bruttomesso D. et al. | 1.9277 (0.7543; 3.1010) | 0.8492 | 0.8496 | 0.4602 | 145.9153 | Yes |
| Omitting Lapolla A. | 1.8337 (0.6314; 3.0360) | 0.8533 | 0.1029 | 0.0108 | 211.9795 | No |
| Omitting Gimenez M. et al. | 2.1295 (0.9653; 3.2938) | 0.8474 | -0.2997 | 0.0861 | 204.0183 | No |
| Omitting Laatikainen L. et al. | 1.9235 (0.7047; 3.1423) | 0.8559 | -0.0197 | 0.0004 | 214.0323 | No |
| Omitting Kallas-Koeman M.M. et al. | 1.7999 (0.5899; 3.0099) | 0.8388 | 0.1167 | 0.0141 | 202.2589 | No |
| Omitting Chico A. et al. | 2.2292 (1.1462; 3.3122) | 0.7122 | -0.4193 | 0.1546 | 129.0283 | No |
| Omitting Burkart W. et al. | 1.9252 (0.7002; 3.1503) | 0.8558 | -0.0243 | 0.0006 | 214.0185 | No |
| Omitting Stella Gonzalez | 1.8012 (0.6025; 2.9998) | 0.8504 | 0.1339 | 0.0183 | 209.6660 | No |

### **Supplementary table 30.** Subgroup analysis Preconceptional HbA1c

| **Variable** | **Subgroup** | **k** | **MD (95% CI)** | **I² (%)** | **p-value (Between Groups)** |
| --- | --- | --- | --- | --- | --- |
| **Risk of Bias** | Serious | 4 | -0.6783 (-0.9663, -0.3903) | 54.4 |  |
|  | Critical | 1 | -0.6800 (-1.0950, -0.2650) | -- |  |
|  | Moderate | 6 | -0.0374 (-0.4148, 0.3400) | 87.6 |  |
|  | Low | 1 | -0.5000 (-1.6615, 0.6615) | -- |  |
|  | **p-value** |  |  |  | 0.0467 |
| **Study Design** | Cohort | 10 | -0.2520 (-0.5511, 0.0470) | 93.2 |  |
|  | RCT | 1 | -0.5000 (-1.6615, 0.6615) | -- |  |
|  | Case-Control | 1 | -0.8800 (-1.2068, -0.5532) | -- |  |
|  | **p-value** |  |  |  | 0.0210 |
| **Time Started** | Before | 10 | -0.2752 (-0.5891, 0.0388) | 93.3 |  |
|  | Both | 1 | -0.6800 (-1.0950, -0.2650) | -- |  |
|  | During | 1 | -0.5000 (-1.6615, 0.6615) | -- |  |
|  | **p-value** |  |  |  | 0.3102 |

### **Supplementary table 31.** Sensitivity analysis Preconceptional HbA1c

| **Author** | **Effect (95% CI: Lower; Upper)** | **I²** | **DFFITS** | **Cook's D** | **QE (del)** | **Is Influential** |
| --- | --- | --- | --- | --- | --- | --- |
| Omitting Mello G. et al | -0.3084 (-0.6055; -0.0112) | 0.9251 | -0.0972 | 0.0098 | 133.5021 | No |
| Omitting Kekalainen P. et al | -0.2861 (-0.5864; 0.0142) | 0.9252 | -0.2493 | 0.0649 | 133.6688 | No |
| Omitting Jotic A. et al | -0.2625 (-0.5536; 0.0285) | 0.8460 | -0.4320 | 0.1758 | 64.9492 | No |
| Omitting Chen R. et al. | -0.3321 (-0.6371; -0.0271) | 0.9227 | 0.0642 | 0.0044 | 129.3089 | No |
| Omitting Talaviya A. et al. | -0.4284 (-0.6738; -0.1829) | 0.7731 | 0.9315 | 0.5467 | 44.0754 | Yes |
| Omitting Abell S.K. et al. | -0.3509 (-0.6525; -0.0493) | 0.9182 | 0.1930 | 0.0392 | 122.3074 | No |
| Omitting Bruttomesso D. et al. | -0.3085 (-0.6129; -0.0042) | 0.9247 | -0.0943 | 0.0096 | 132.7988 | No |
| Omitting Lapolla A. | -0.3809 (-0.6578; -0.1040) | 0.9183 | 0.4214 | 0.1661 | 122.4658 | No |
| Omitting Gimenez M. et al. | -0.3554 (-0.6496; -0.0612) | 0.9213 | 0.2278 | 0.0527 | 127.0294 | No |
| Omitting Kallas-Koeman M.M. et al. | -0.2891 (-0.5949; 0.0166) | 0.9247 | -0.2248 | 0.0545 | 132.8146 | No |
| Omitting Donald R. Coustan | -0.3144 (-0.6063; -0.0225) | 0.9251 | -0.0564 | 0.0032 | 133.5635 | No |
| Omitting Stella Gonzalez | -0.2632 (-0.5502; 0.0237) | 0.9246 | -0.4277 | 0.1716 | 132.6392 | No |

**Supplementary figure 1.** ROBINS-I Tool for non-randomised studies of intervention


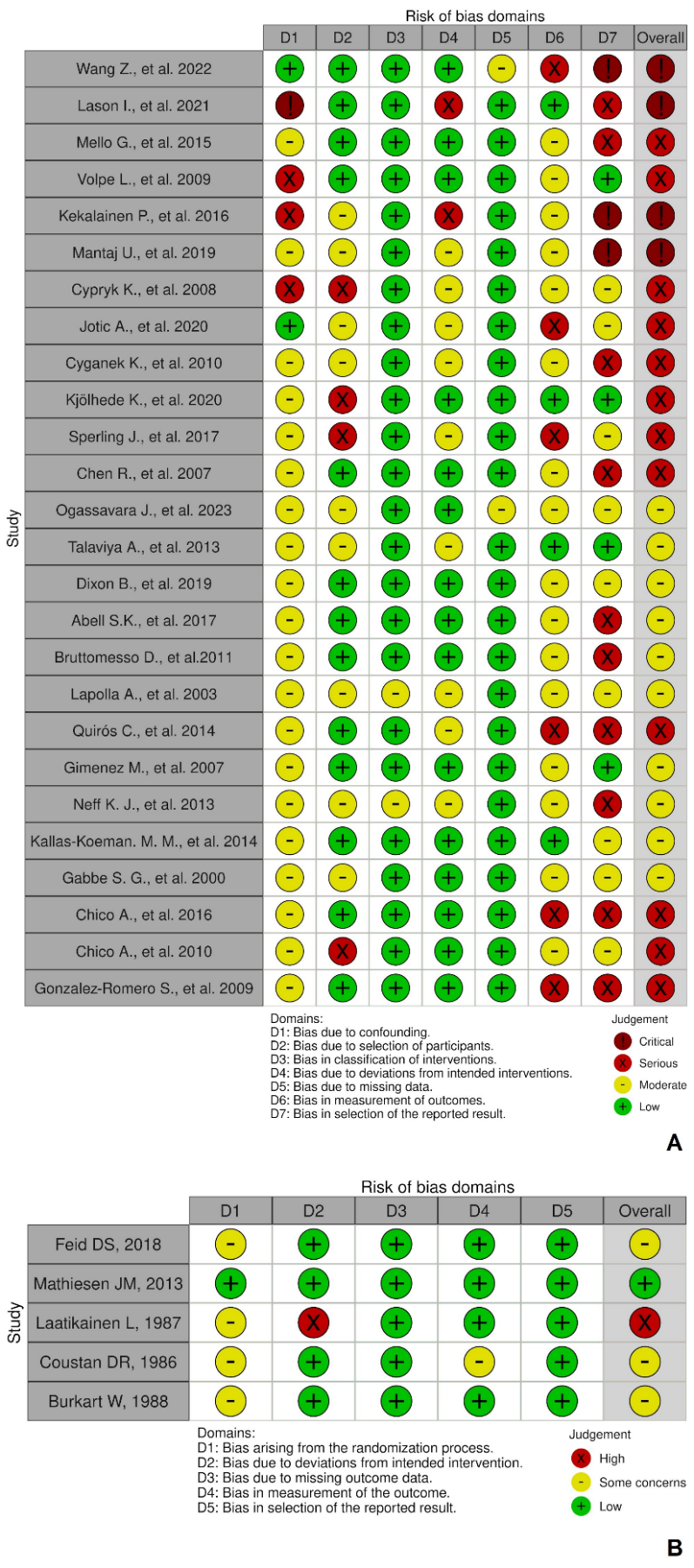


### **Supplementary figure 2.** Forest Plot Preeclampsia and Bleeding


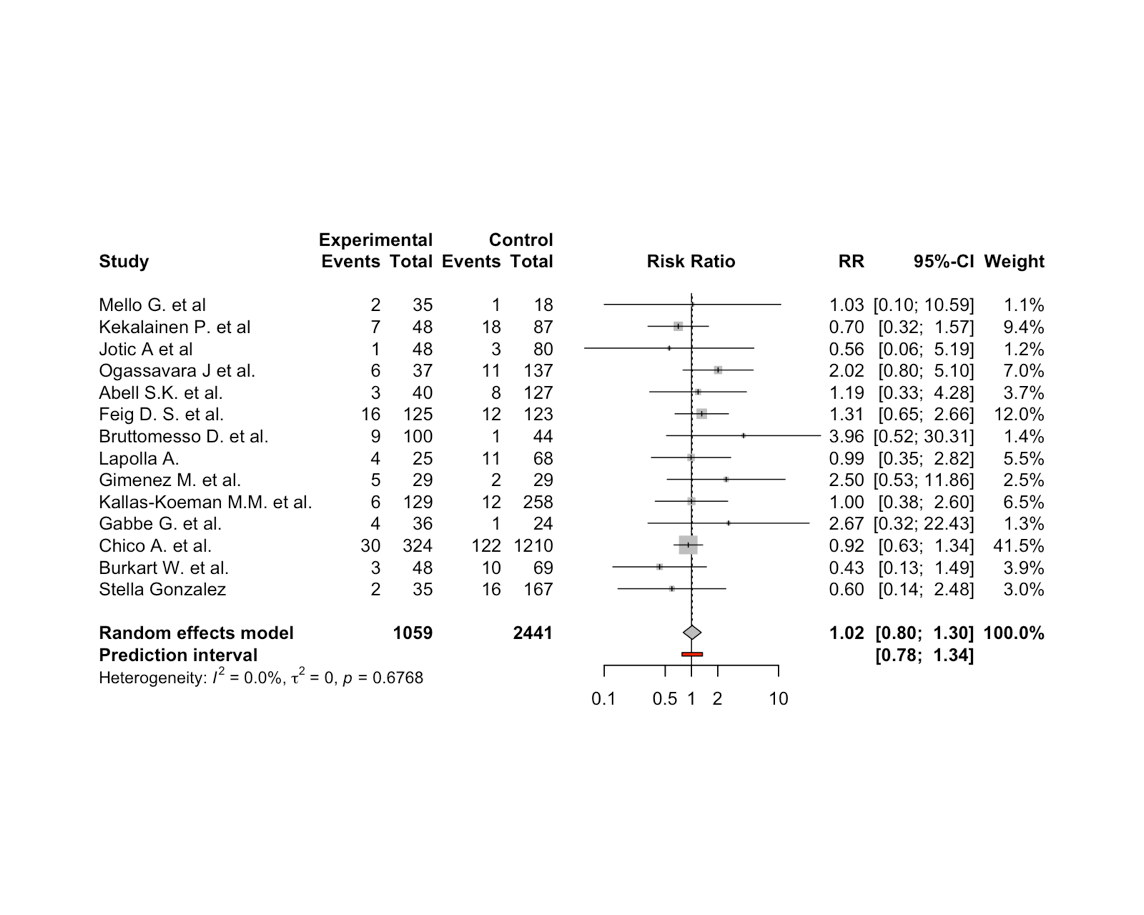


A forest plot illustrating the RR (relative risk) and CI (confidence interval) of preeclampsia and bleeding events in pregnant patients with type 1 diabetes treated with multiple daily insulin injections across fourteen studies under a random effects model showed low heterogeneity among studies (I^2^ =0%).

### **Supplementary figure 3.** Funnel Plot Preeclampsia and Bleeding


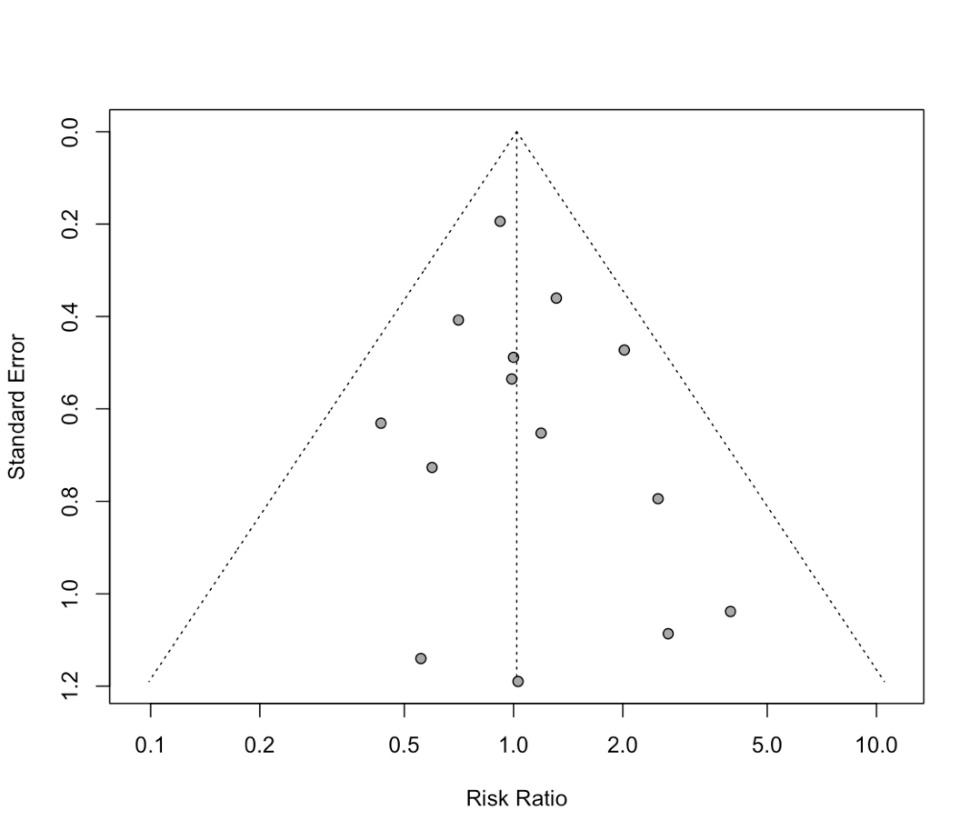


Funnel plot showing a symmetric distribution of studies, suggesting no significant publication bias

**Supplementary figure 4.** Forest Plot Cesarean Delivery


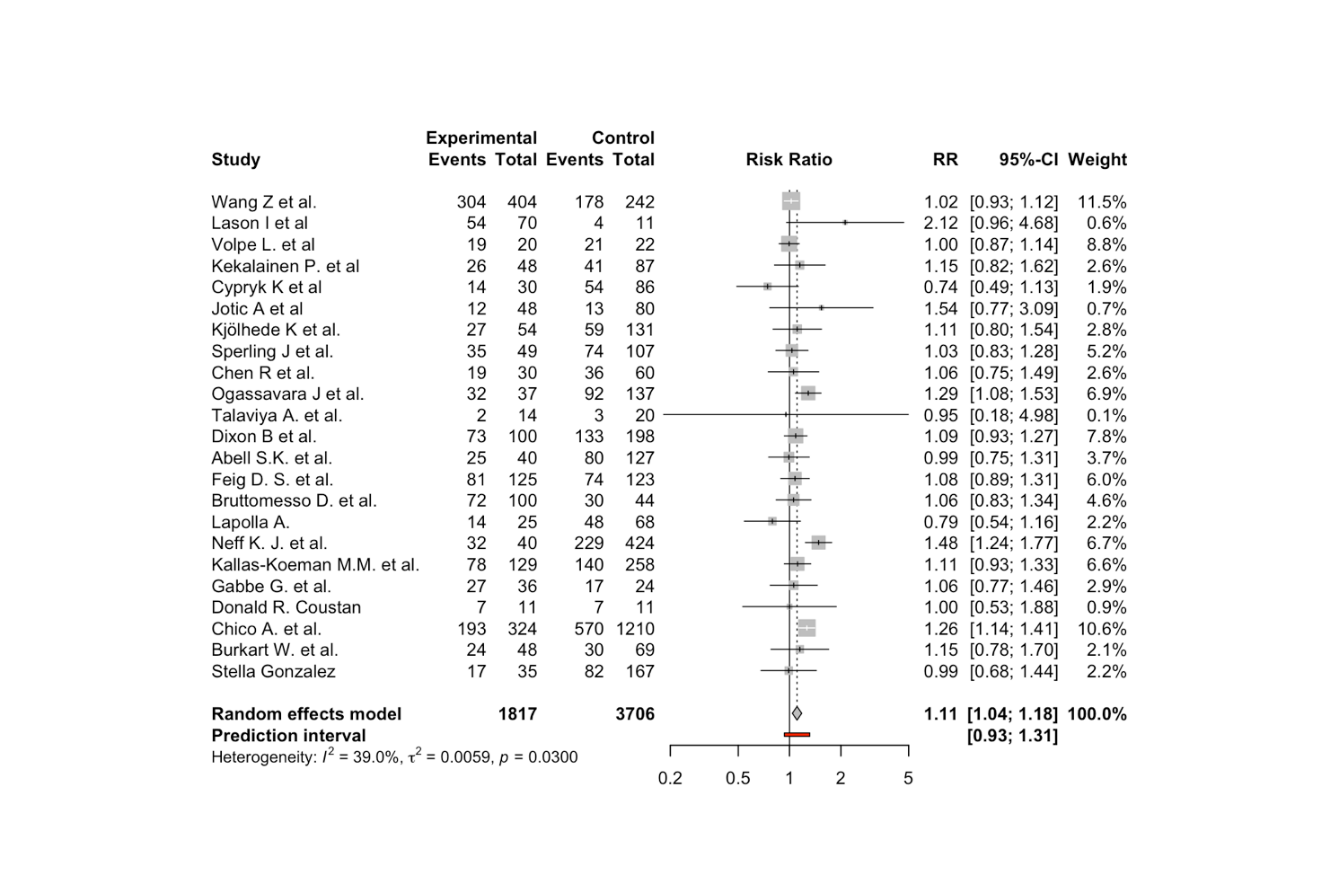


A forest plot illustrating the RR (relative risk) and CI (confidence interval) of cesarean delivery in pregnant patients with type 1 diabetes treated with multiple daily insulin injections across twenty-three studies under a random effects model showed moderate heterogeneity among studies (I^2^ =39%).

**Supplementary figure 5.** Funnel Plot Cesarean Delivery


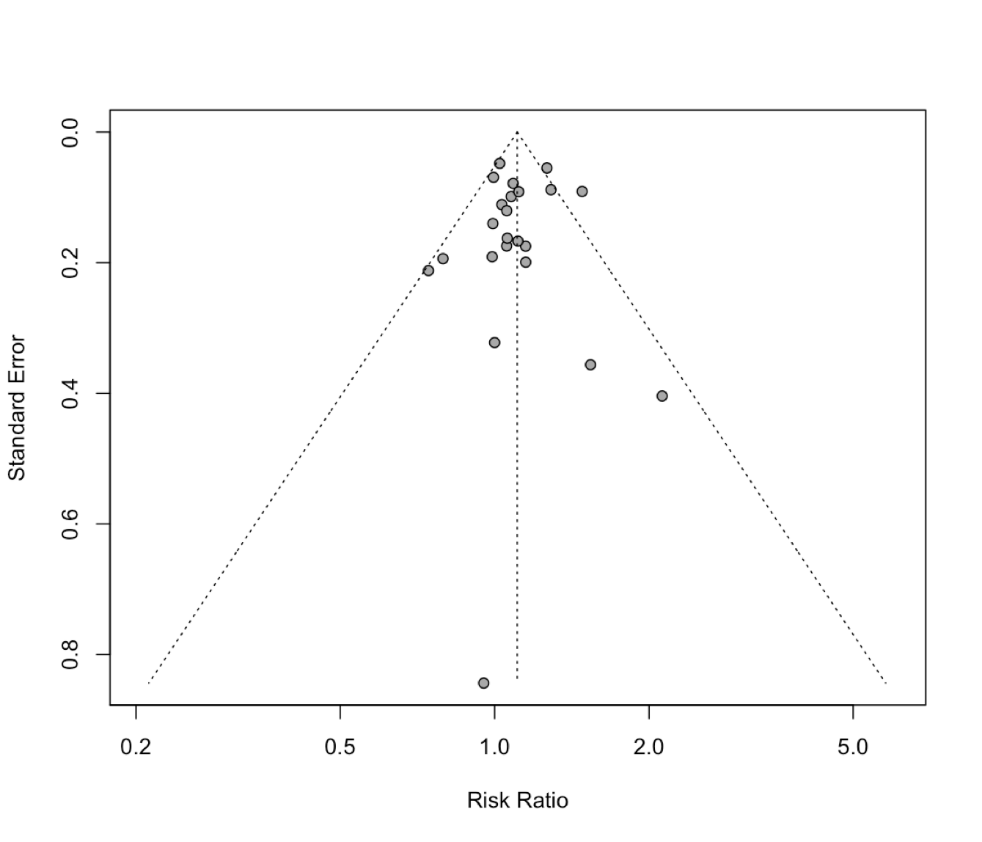


Funnel plot showing an asymmetric distribution of studies, which may indicate potential publication bias

**Supplementary figure 6.** Forest Plot Congenital Malformations


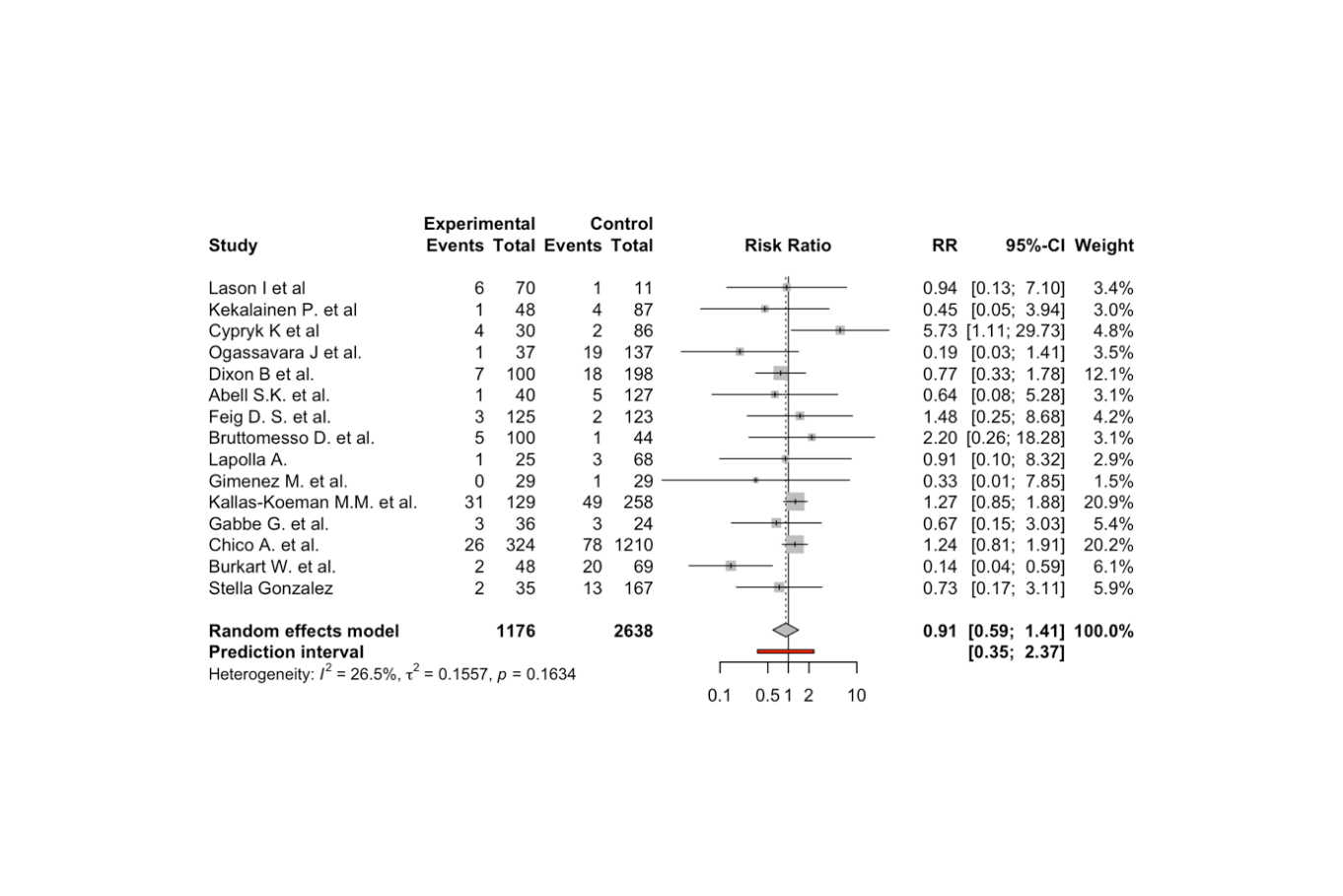


A forest plot illustrating the RR (relative risk) and CI (confidence interval) of congenital malformations in pregnant patients with type 1 diabetes treated with multiple daily insulin injections across fifth teen studies under a random effects model showed moderate heterogeneity among studies (I^2^ =26.5%).

### **Supplementary figure 7.** Funnel Plot Congenital Malformations


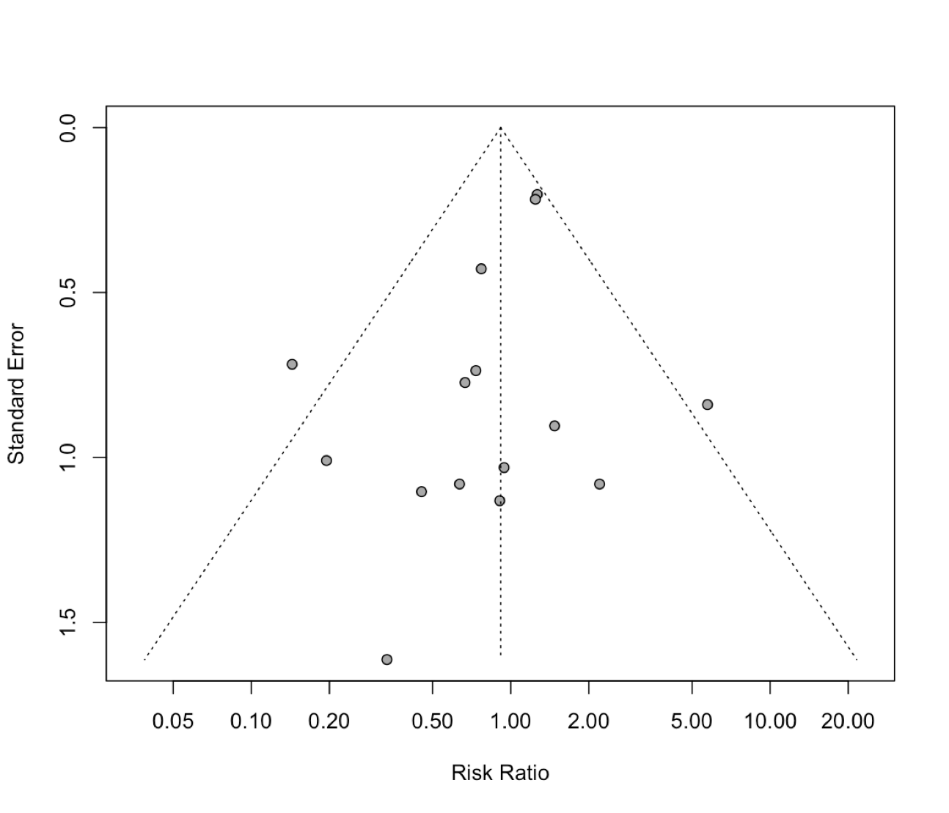


Funnel plot showing an asymmetric distribution of studies, which may indicate potential publication bias

**Supplementary figure 8.** Forest Plot Preterm Birth


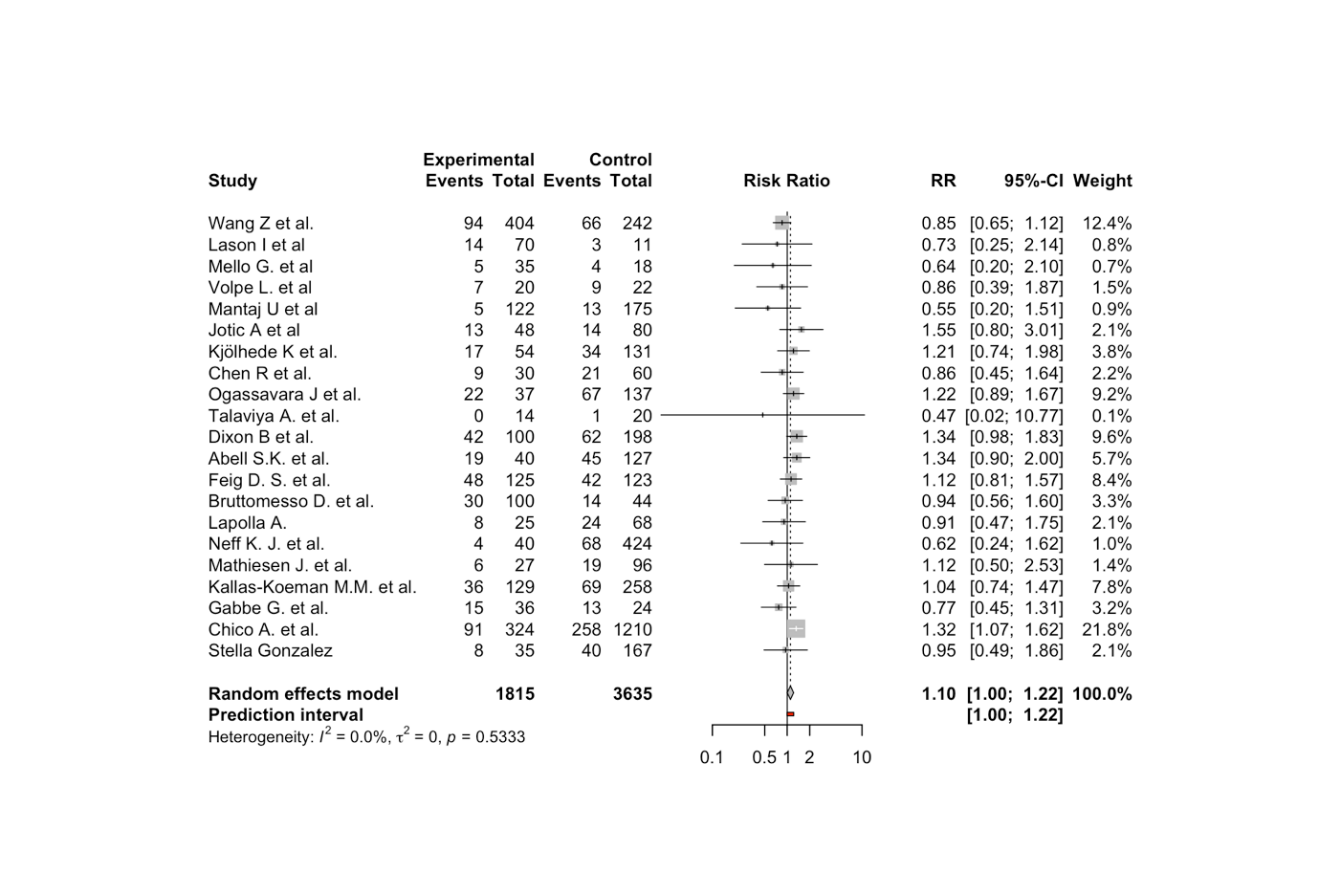


A forest plot illustrating the RR (relative risk) and CI (confidence interval) of preterm birth in pregnant patients with type 1 diabetes treated with multiple daily insulin injections across twenty-one studies under a random effects model showed low heterogeneity among studies (I^2^ =0%).

### **Supplementary figure 9.** Funnel Plot Preterm Birth


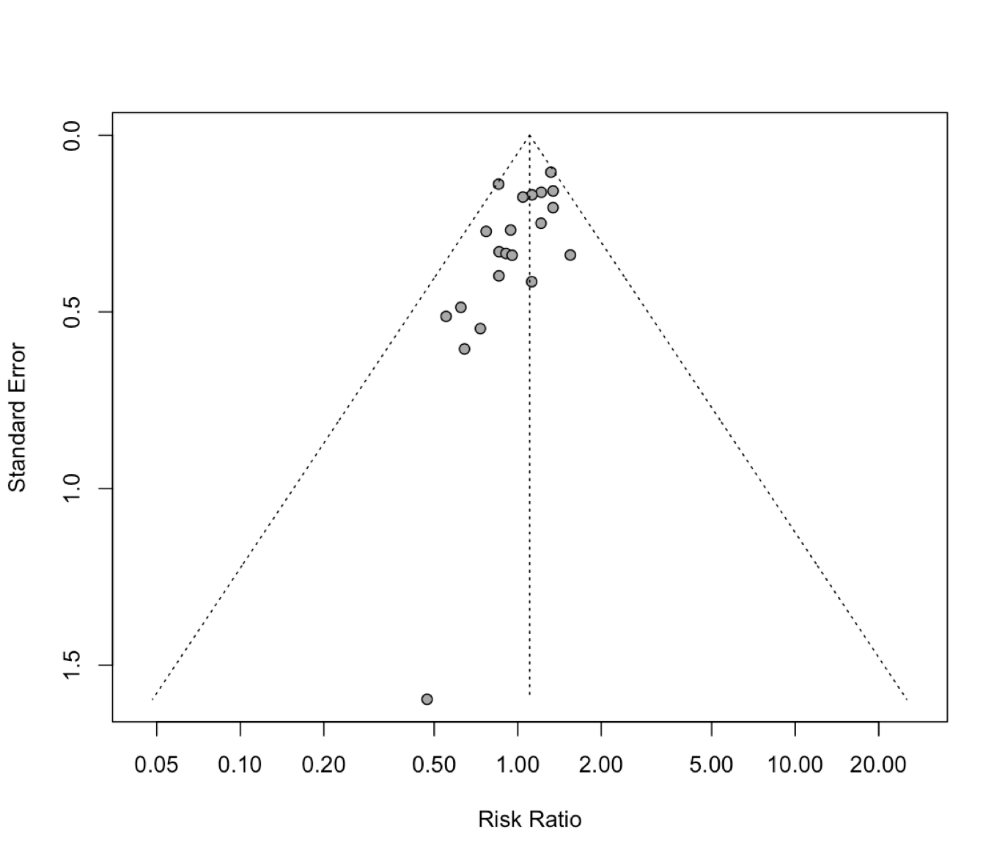


Funnel plot showing a symmetric distribution of studies, suggesting no significant publication bias

**Supplementary figure 10.** Forest Plot Large for Gestational Age


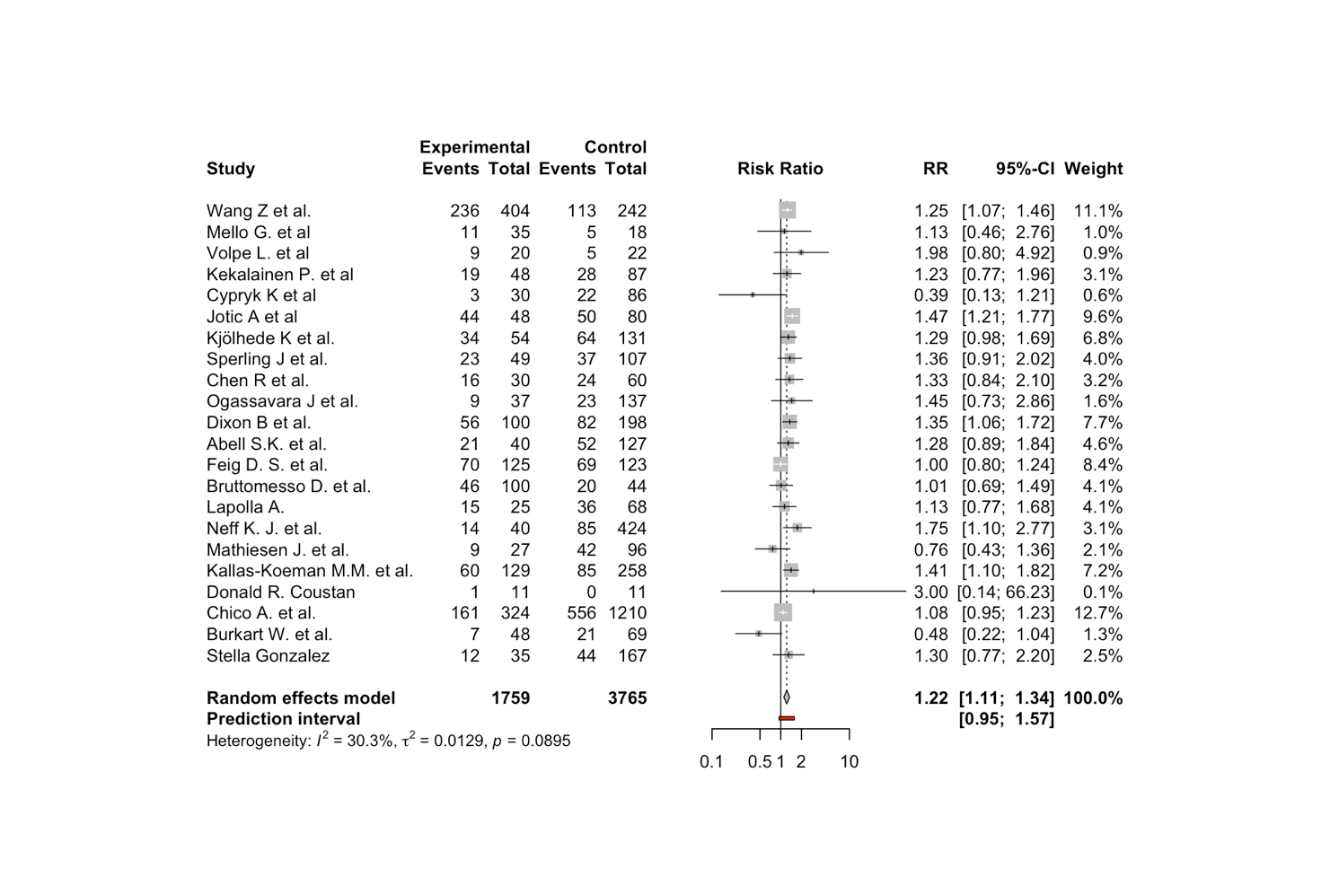


A forest plot illustrating the RR (relative risk) and CI (confidence interval) of large for gestational age in pregnant patients with type 1 diabetes treated with multiple daily insulin injections across twenty-two studies under a random effects model showed moderate heterogeneity among studies (I^2^ =30.3%).

**Supplementary figure 11.** Funnel Plot Large for Gestational Age


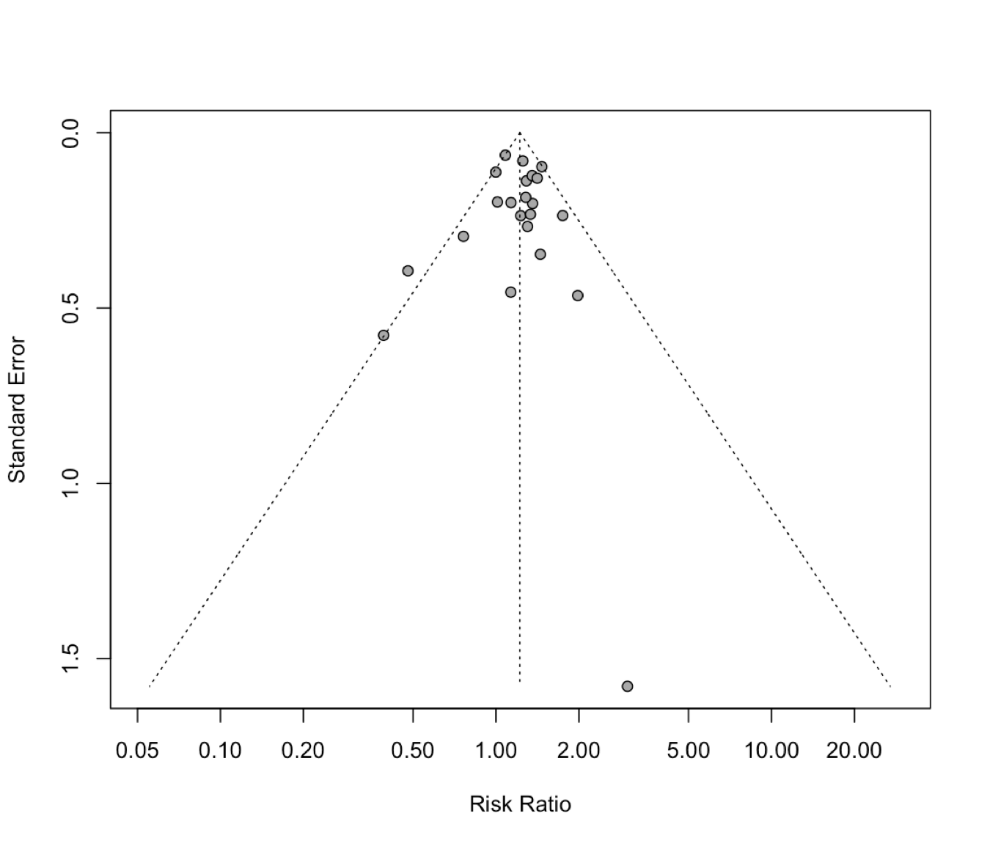


Funnel plot showing an asymmetric distribution of studies, which may indicate potential publication bias

**Supplementary figure 12.** Forest Plot Small for Gestational Age


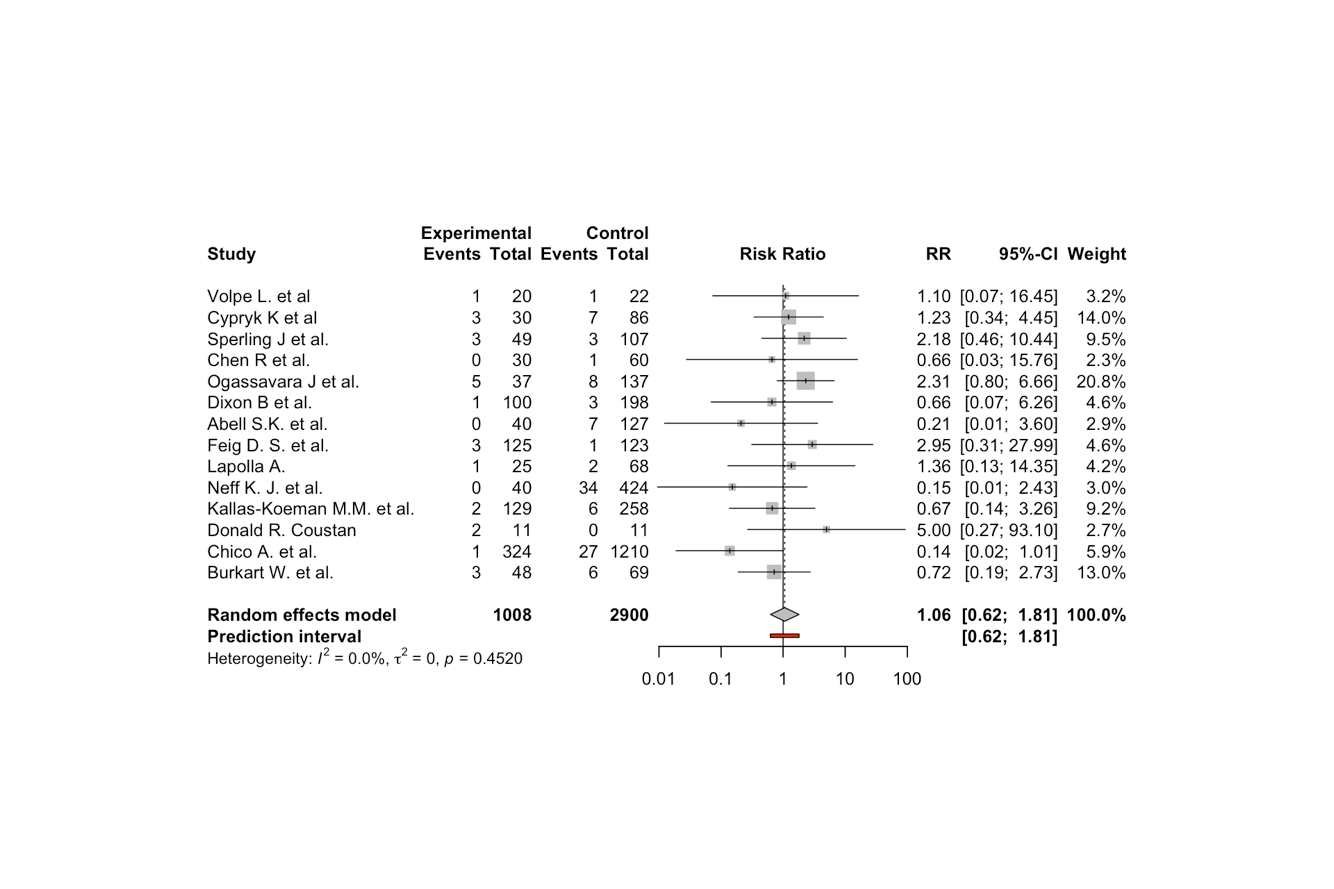


A forest plot illustrating the RR (relative risk) and CI (confidence interval) of small for gestational age in pregnant patients with type 1 diabetes treated with multiple daily insulin injections across fourteen studies under a random effects model showed low heterogeneity among studies (I^2^ =0%).

### **Supplementary figure 13.** Funnel Plot Small for Gestational Age


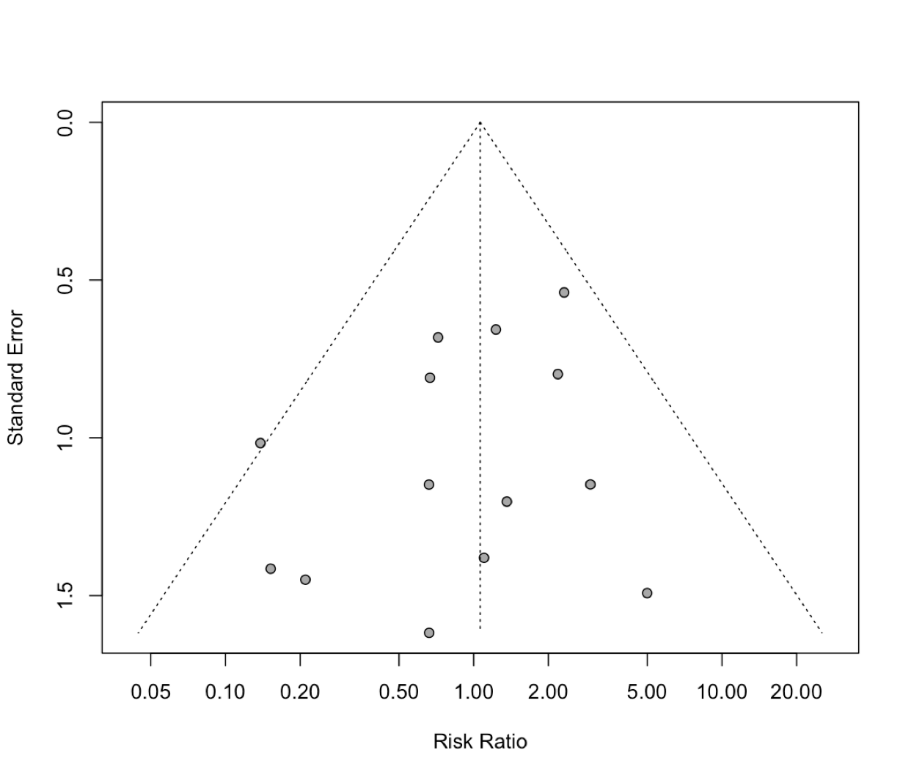


Funnel plot showing an asymmetric distribution of studies, which may indicate potential publication bias

**Supplementary figure 14.** Forest Plot Neonatal Hypoglycemia


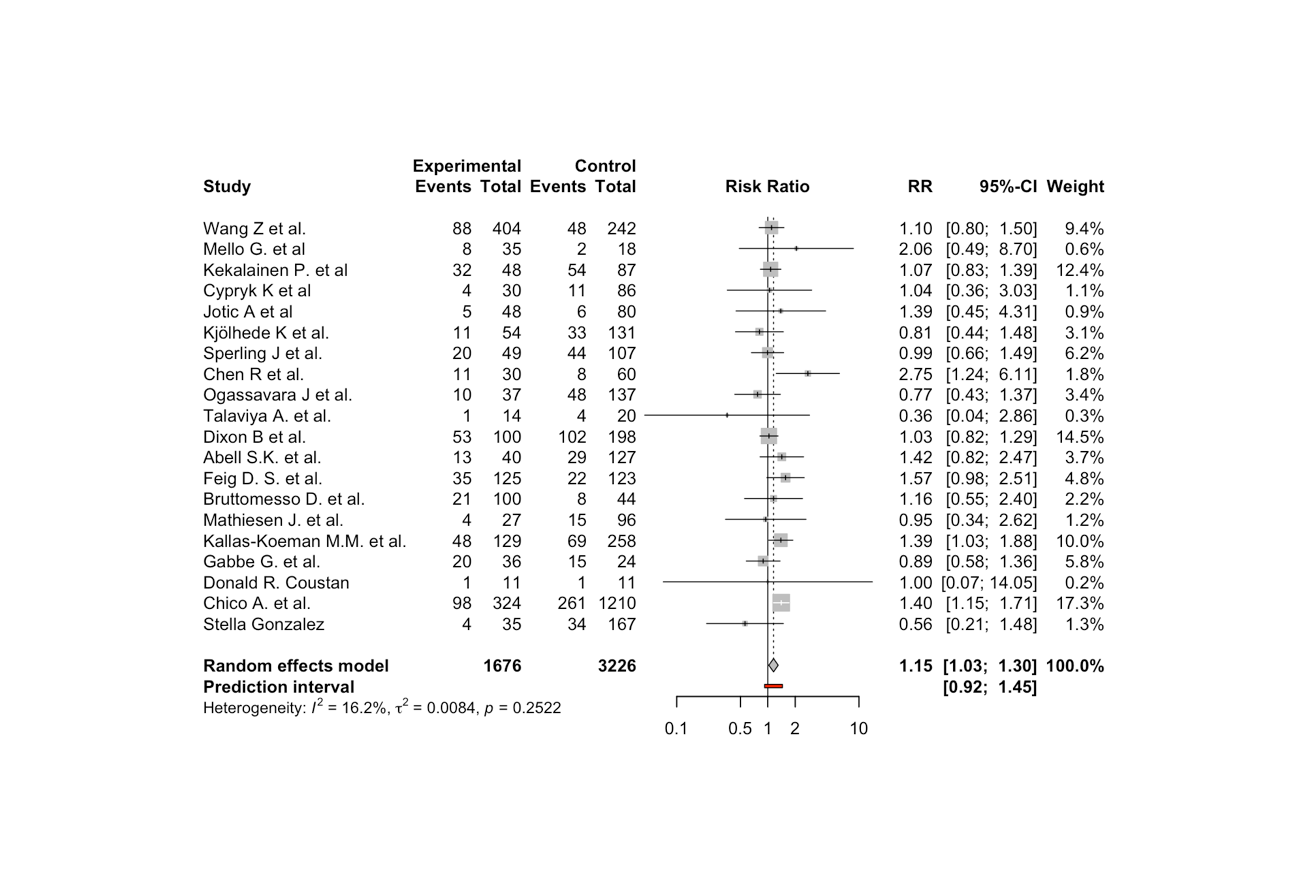


A forest plot illustrating the RR (relative risk) and CI (confidence interval) of neonatal hypoglycemia in pregnant patients with type 1 diabetes treated with multiple daily insulin injections across twenty studies under a random effects model showed low heterogeneity among studies (I^2^ =16.2%).

**Supplementary figure 15.** Funnel Plot Neonatal Hypoglycemia


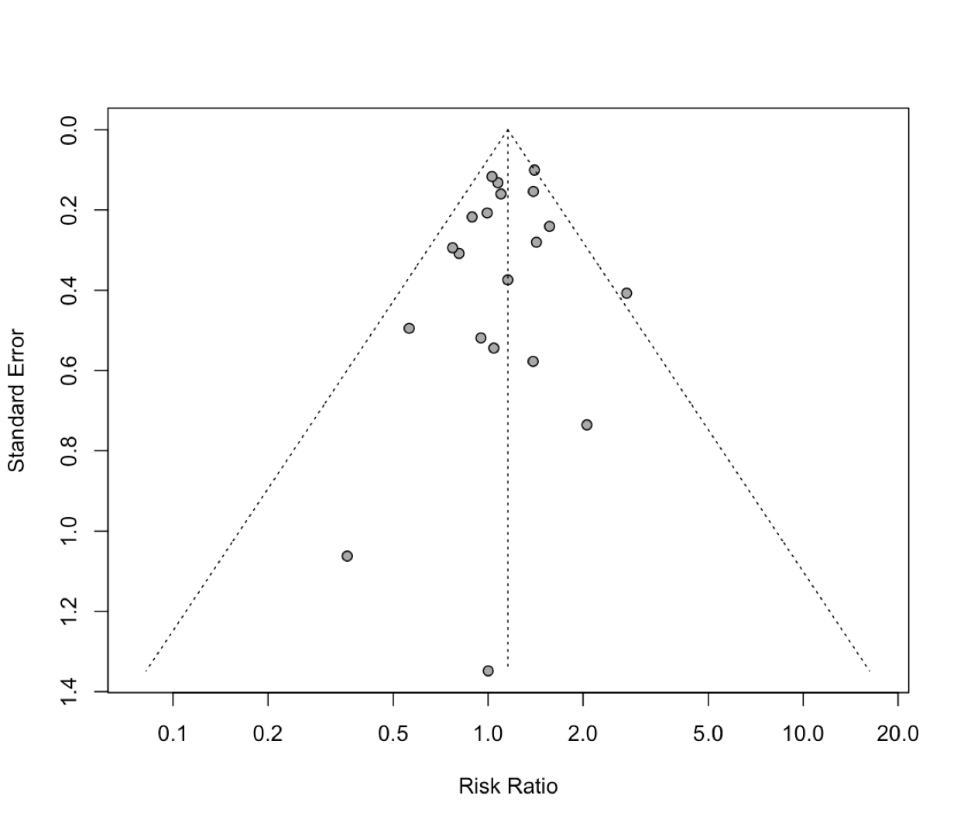


Funnel plot showing an asymmetric distribution of studies, which may indicate potential publication bias

**Supplementary figure 16.** Forest Plot Neonatal Jaundice


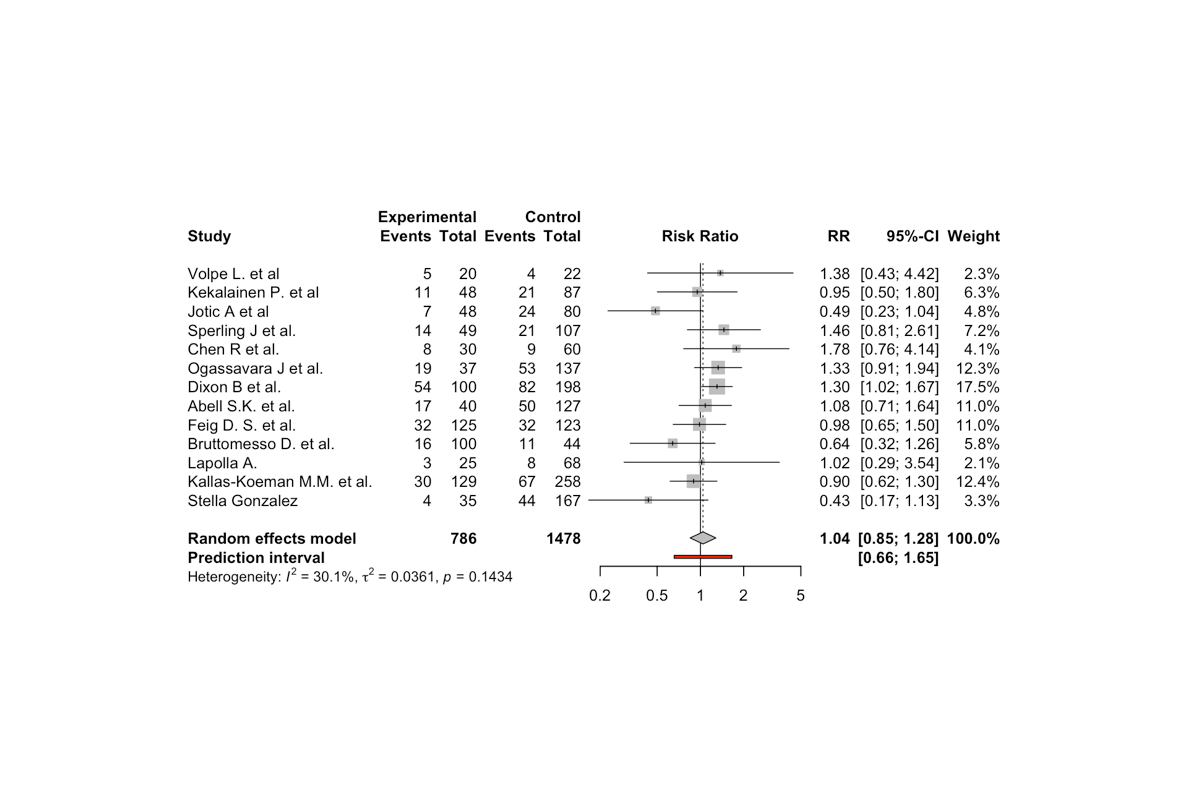


A forest plot illustrating the RR (relative risk) and CI (confidence interval) of neonatal jaundice in pregnant patients with type 1 diabetes treated with multiple daily insulin injections across thirteen studies under a random effects model showed moderate heterogeneity among studies (I^2^ =30.1%).

**Supplementary figure 17.** Funnel Plot Neonatal Jaundice


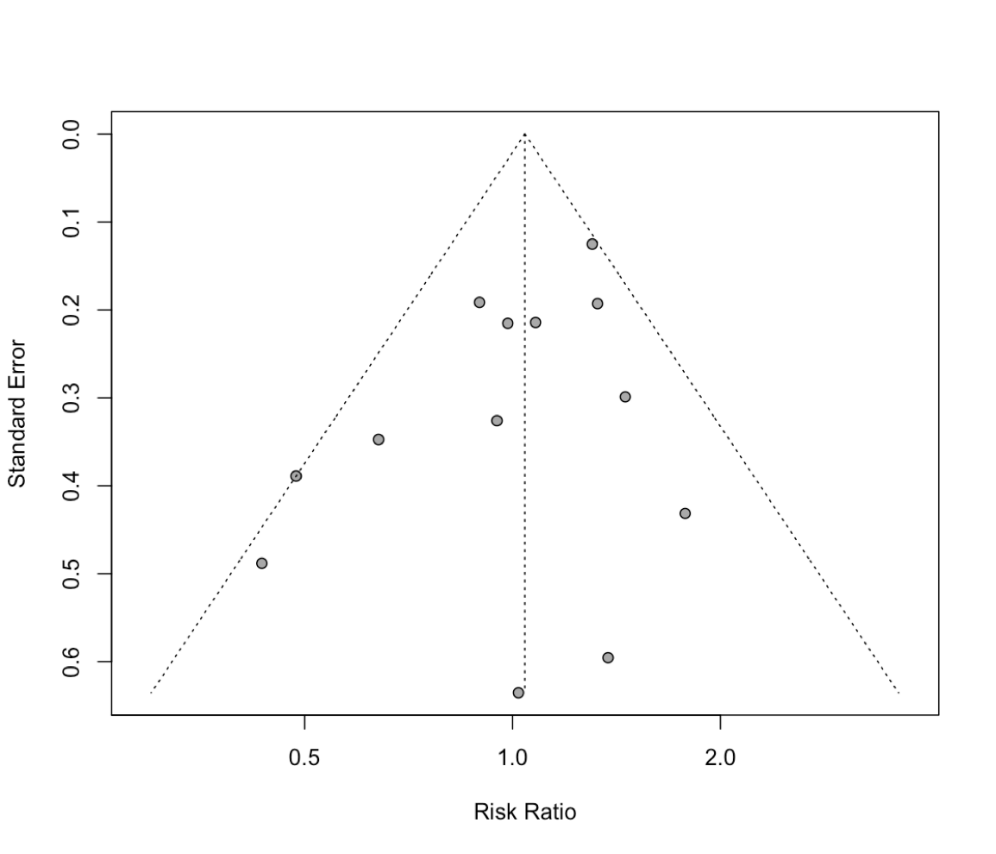


Funnel plot showing a symmetric distribution of studies, suggesting no significant publication bias

**Supplementary figure 18.** Forest Plot Birth Weight


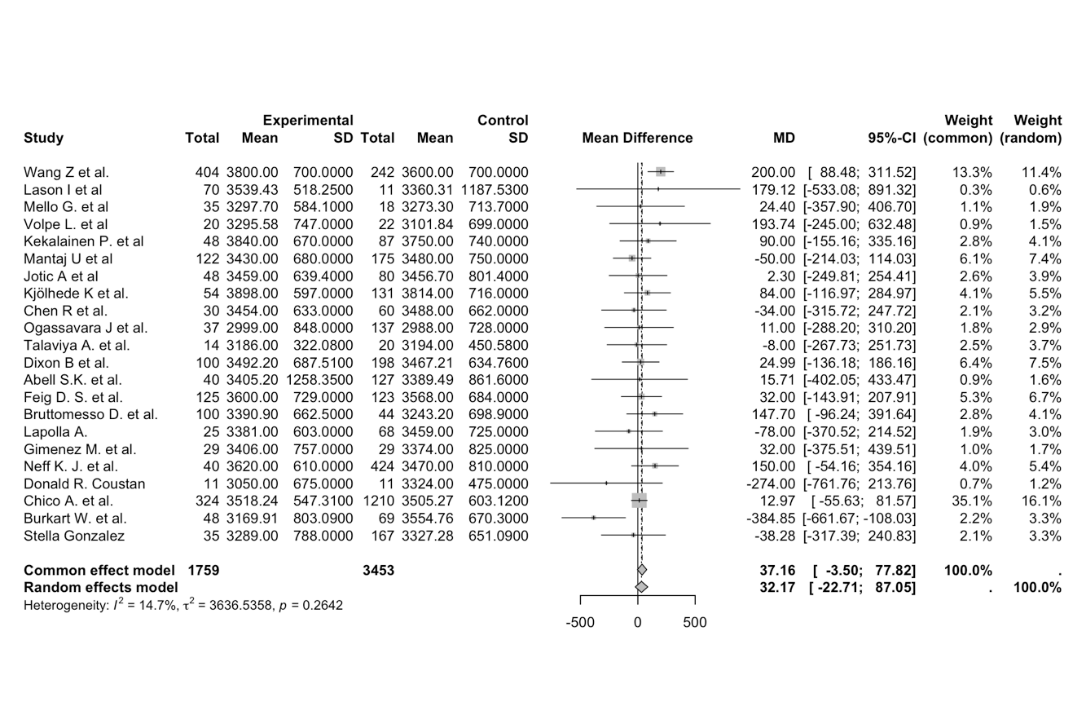


A forest plot illustrating the MD (mean difference) and CI (confidence interval) of birth weight in pregnant patients with type 1 diabetes treated with multiple daily insulin injections across twenty-two studies under a random effects model showed low heterogeneity among studies (I^2^ =14.7%).

**Supplementary figure 19.** Funnel Plot Birth Weight


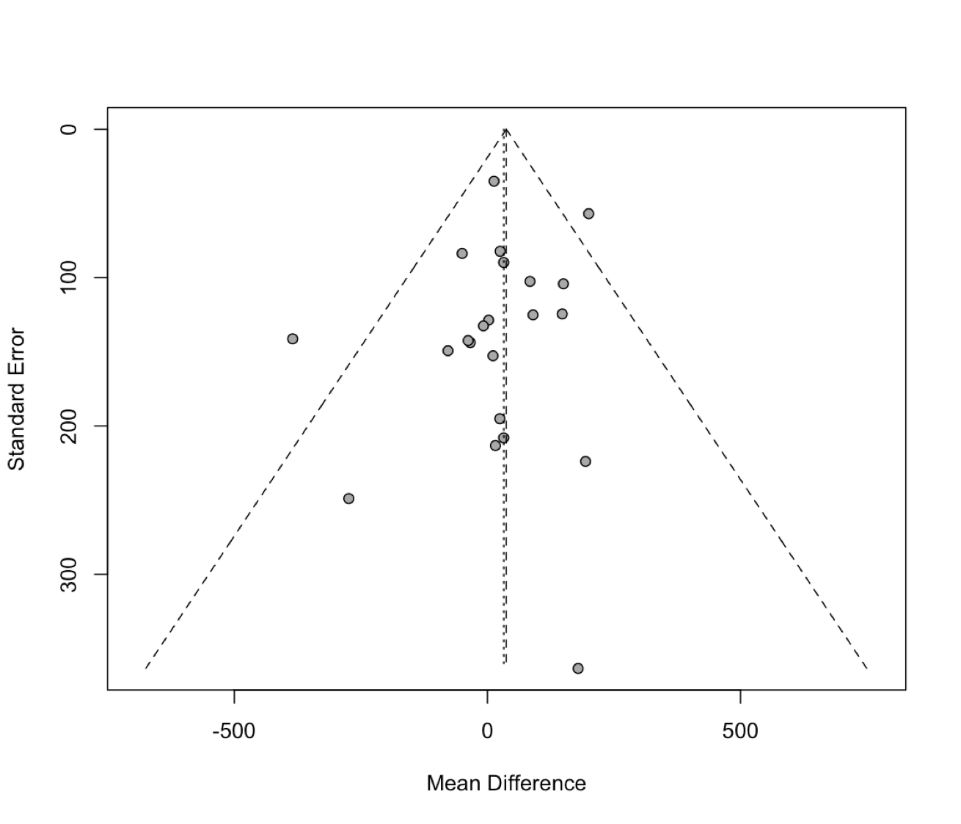


Funnel plot showing an asymmetric distribution of studies, which may indicate potential publication bias

**Supplementary figure 20.** Forest Plot Weight Gain


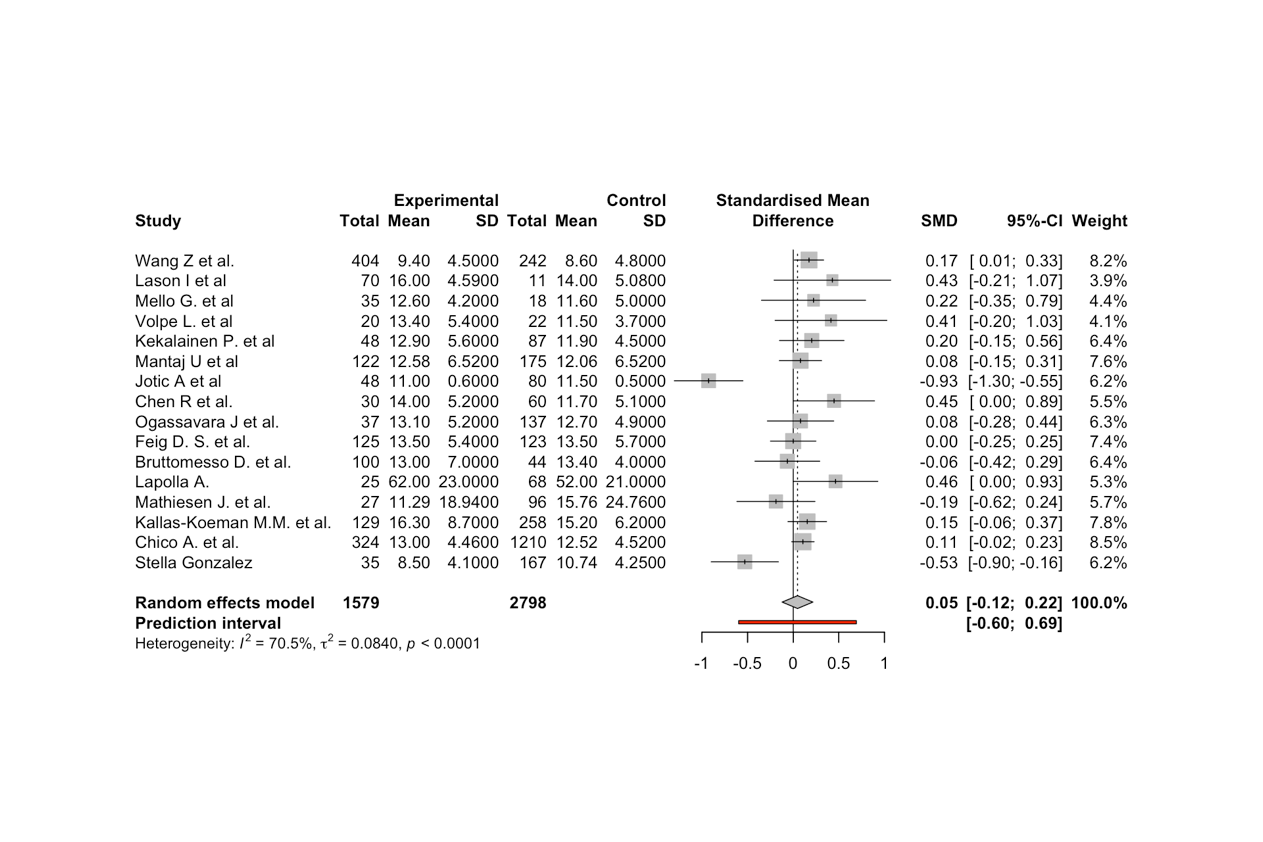


A forest plot illustrating the SMD (standard mean difference) and CI (confidence interval) of weight gain in pregnant patients with type 1 diabetes treated with multiple daily insulin injections across sixteen studies under a random effects model showed large heterogeneity among studies (I^2^ =70.5%).

**Supplementary figure 21.** Funnel Plot Weight Gain


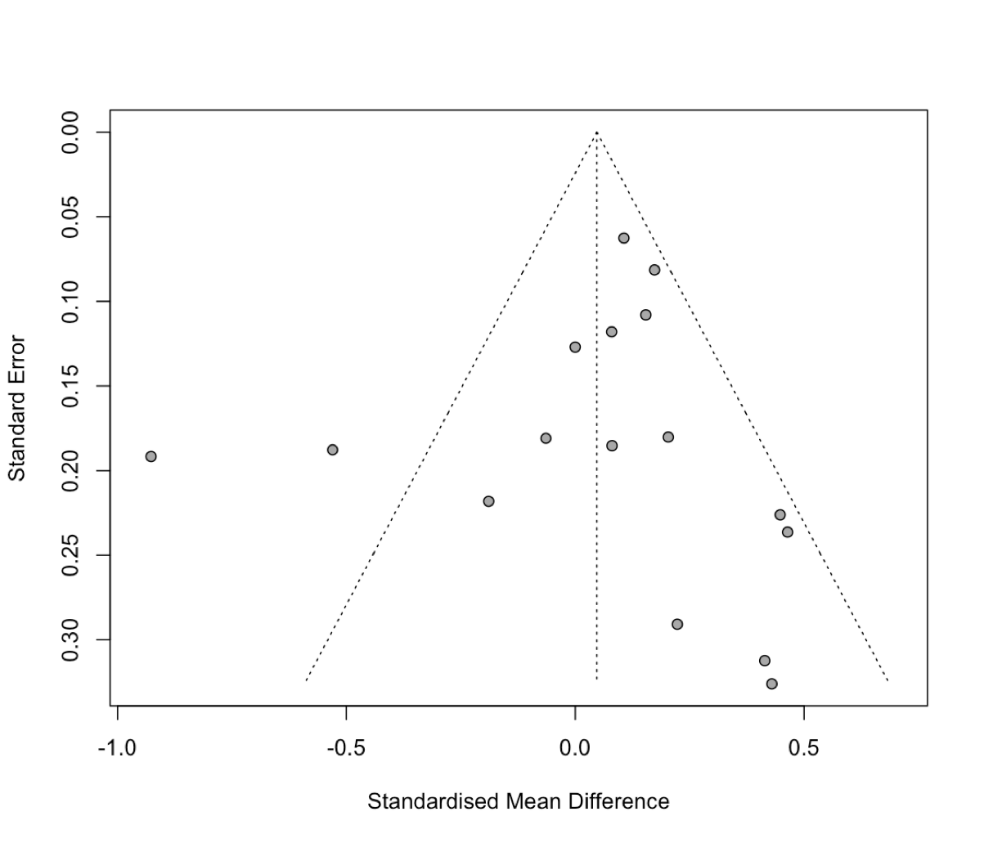


Funnel plot showing an asymmetric distribution of studies, which may indicate potential publication bias

**Supplementary figure 22.** Forest Plot Time with Diabetes


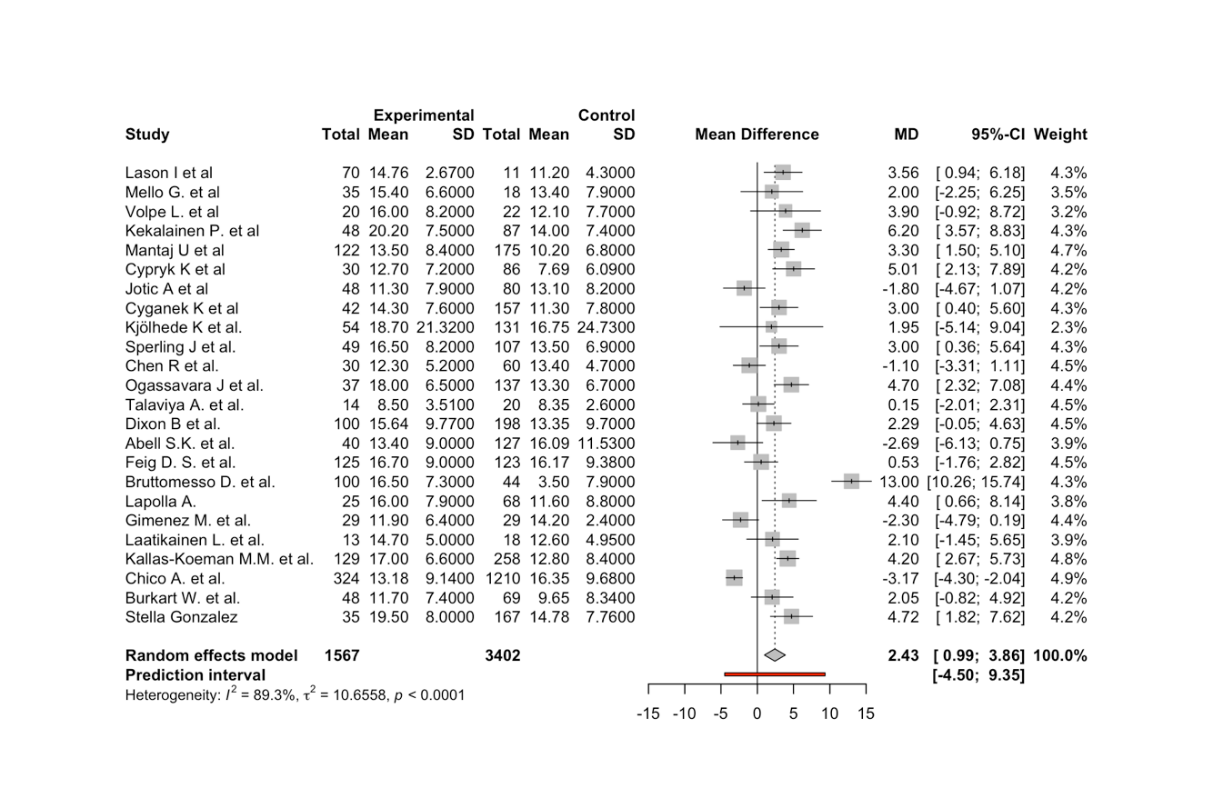


A forest plot illustrating the MD (mean difference) and CI (confidence interval) of time with diabetes in pregnant patients with type 1 diabetes treated with multiple daily insulin injections across twenty-four studies under a random effects model showed large heterogeneity among studies (I^2^ =89.3%).

**Supplementary figure 23.** Funnel Plot Time with Diabetes


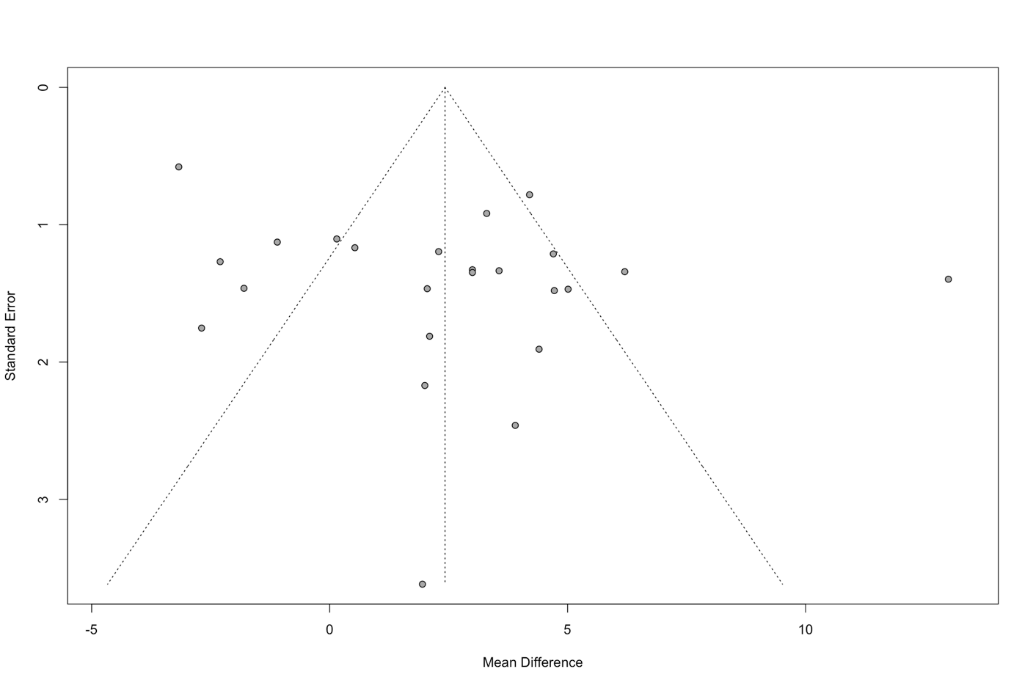


Funnel plot showing an asymmetric distribution of studies, which may indicate potential publication bias

**PRISMA 2020 CHECKLIST**

| **Section and Topic** | **Item #** | **Checklist item** | **Location where item is reported** |
| --- | --- | --- | --- |
| **TITLE** | | |  |
| Title | 1 | Identify the report as a systematic review. | Page 1 |
| **ABSTRACT** | | |  |
| Abstract | 2 | See the PRISMA 2020 for Abstracts checklist. | Page 2-3 |
| **INTRODUCTION** | | |  |
| Rationale | 3 | Describe the rationale for the review in the context of existing knowledge. | Page 4-5 |
| Objectives | 4 | Provide an explicit statement of the objective(s) or question(s) the review addresses. | Page 4-5 |
| **METHODS** | | |  |
| Eligibility criteria | 5 | Specify the inclusion and exclusion criteria for the review and how studies were grouped for the syntheses. | Page 6 |
| Information sources | 6 | Specify all databases, registers, websites, organisations, reference lists and other sources searched or consulted to identify studies. Specify the date when each source was last searched or consulted. | Page 6 |
| Search strategy | 7 | Present the full search strategies for all databases, registers and websites, including any filters and limits used. | Page 6 |
| Selection process | 8 | Specify the methods used to decide whether a study met the inclusion criteria of the review, including how many reviewers screened each record and each report retrieved, whether they worked independently, and if applicable, details of automation tools used in the process. | Page 6 |
| Data collection process | 9 | Specify the methods used to collect data from reports, including how many reviewers collected data from each report, whether they worked independently, any processes for obtaining or confirming data from study investigators, and if applicable, details of automation tools used in the process. | Page 6-7 |
| Data items | 10a | List and define all outcomes for which data were sought. Specify whether all results that were compatible with each outcome domain in each study were sought (e.g. for all measures, time points, analyses), and if not, the methods used to decide which results to collect. | Page 6 |
|  | 10b | List and define all other variables for which data were sought (e.g. participant and intervention characteristics, funding sources). Describe any assumptions made about any missing or unclear information. | Page 6 |
| Study risk of bias assessment | 11 | Specify the methods used to assess risk of bias in the included studies, including details of the tool(s) used, how many reviewers assessed each study and whether they worked independently, and if applicable, details of automation tools used in the process. | Page 6 |
| Effect measures | 12 | Specify for each outcome the effect measure(s) (e.g. risk ratio, mean difference) used in the synthesis or presentation of results. | Page 6-7 |
| Synthesis methods | 13a | Describe the processes used to decide which studies were eligible for each synthesis (e.g. tabulating the study intervention characteristics and comparing against the planned groups for each synthesis (item #5)). | Page 6 |
|  | 13b | Describe any methods required to prepare the data for presentation or synthesis, such as handling of missing summary statistics, or data conversions. | N/A |
|  | 13c | Describe any methods used to tabulate or visually display results of individual studies and syntheses. | N/A |
|  | 13d | Describe any methods used to synthesize results and provide a rationale for the choice(s). If meta-analysis was performed, describe the model(s), method(s) to identify the presence and extent of statistical heterogeneity, and software package(s) used. | Page 6-7 |
|  | 13e | Describe any methods used to explore possible causes of heterogeneity among study results (e.g. subgroup analysis, meta-regression). | Page 6 |
|  | 13f | Describe any sensitivity analyses conducted to assess robustness of the synthesized results. | Page 6 |
| Reporting bias assessment | 14 | Describe any methods used to assess risk of bias due to missing results in a synthesis (arising from reporting biases). | Page 6 |
| Certainty assessment | 15 | Describe any methods used to assess certainty (or confidence) in the body of evidence for an outcome. | Page 6 |
| **RESULTS** | | |  |
| Study selection | 16a | Describe the results of the search and selection process, from the number of records identified in the search to the number of studies included in the review, ideally using a flow diagram. | Page 7 |
|  | 16b | Cite studies that might appear to meet the inclusion criteria, but which were excluded, and explain why they were excluded. | Page 7 |
| Study characteristics | 17 | Cite each included study and present its characteristics. | Page 26-31 |
| Risk of bias in studies | 18 | Present assessments of risk of bias for each included study. | Supplemental  Material |
| Results of individual studies | 19 | For all outcomes, present, for each study: (a) summary statistics for each group (where appropriate) and (b) an effect estimate and its precision (e.g. confidence/credible interval), ideally using structured tables or plots. | Page 32 |
| Results of syntheses | 20a | For each synthesis, briefly summarise the characteristics and risk of bias among contributing studies. | Page 7-8 |
|  | 20b | Present results of all statistical syntheses conducted. If meta-analysis was done, present for each the summary estimate and its precision (e.g. confidence/credible interval) and measures of statistical heterogeneity. If comparing groups, describe the direction of the effect. | Page 8-15 |
|  | 20c | Present results of all investigations of possible causes of heterogeneity among study results. | Page 8-15 |
|  | 20d | Present results of all sensitivity analyses conducted to assess the robustness of the synthesized results. | Supplemental  Material |
| Reporting biases | 21 | Present assessments of risk of bias due to missing results (arising from reporting biases) for each synthesis assessed. | Supplemental  Material |
| Certainty of evidence | 22 | Present assessments of certainty (or confidence) in the body of evidence for each outcome assessed. | Page 32 |
| **DISCUSSION** | | |  |
| Discussion | 23a | Provide a general interpretation of the results in the context of other evidence. | Page 15-16 |
|  | 23b | Discuss any limitations of the evidence included in the review. | Page 17 |
|  | 23c | Discuss any limitations of the review processes used. | Page 17 |
|  | 23d | Discuss implications of the results for practice, policy, and future research. | Page 17-18 |
| **OTHER INFORMATION** | | |  |
| Registration and protocol | 24a | Provide registration information for the review, including register name and registration number, or state that the review was not registered. | Page 5 |
|  | 24b | Indicate where the review protocol can be accessed, or state that a protocol was not prepared. | Page 5 |
|  | 24c | Describe and explain any amendments to information provided at registration or in the protocol. | N/A |
| Support | 25 | Describe sources of financial or non-financial support for the review, and the role of the funders or sponsors in the review. | Page 19 |
| Competing interests | 26 | Declare any competing interests of review authors. | N/A |
| Availability of data, code and other materials | 27 | Report which of the following are publicly available and where they can be found: template data collection forms; data extracted from included studies; data used for all analyses; analytic code; any other materials used in the review. | Page 6 |
